# Gut microbiota dysbiosis and the gut–lung axis in COPD: mechanisms, clinical relevance, and microbiota-targeted interventions

**DOI:** 10.3389/fcell.2026.1915007

**Published:** 2026-09-02

**Authors:** Yinghui Chai, Junya Lan, Kang Li, Xue Mei, Yanan Chen, Nannan Zhou, Xin Liu, Wenping Gong, Hong Lei

**Affiliations:** 1 Department of Clinical Laboratory, the Eighth Medical Center of PLA General Hospital, Beijing, China; 2 Graduate School, Hebei North University, Zhangjiakou, Hebei, China; 3 Senior Department of Tuberculosis, Chinese PLA General Hospital, Beijing, China

**Keywords:** chronic obstructive pulmonary disease, dysbiosis, gut microbiota, gut–lung axis, microbial metabolites, systemic inflammation

## Abstract

Chronic obstructive pulmonary disease (COPD), which is characterised by persistent inflammation and airflow limitation, has increasingly been linked to the gut-lung axis. Patients with COPD commonly exhibit reduced diversity of gut microbiota, decreased levels of bacteria that produce short-chain fatty acids (SCFAs), increased levels of opportunistic pathogens, and compromised intestinal barrier function. These alterations are driven by factors such as smoking, hypoxia, oxidative stress, medication use and ageing, and promote bacterial translocation and systemic inflammation, thereby exacerbating lung injury. Gut microbiota metabolites, including SCFAs, bile acids, tryptophan metabolites and trimetlylamine N-oxide (TMAO), further modulate immune responses and metabolic pathways, thereby influencing disease progression. Intervention strategies targeting the microbiome, including dietary fibre, probiotics, prebiotics, faecal microbiota transplantation (FMT) and phage therapy, have demonstrated potential therapeutic value, though clinical evidence remains limited. Elucidating the mechanisms linking gut dysbiosis and COPD will provide novel targets for precision interventions and disease management.

## 1 Introduction

Chronic obstructive pulmonary disease (COPD) is a significant global health issue that poses a serious challenge to public health. The primary characteristic of COPD is airflow limitation, which is usually progressive and irreversible (Liu C, et al., 2022). As the global population ages, COPD-related frailty has also become a hot topic in research. Despite advances in current therapeutic strategies, available treatments mainly alleviate symptoms and reduce exacerbations but have limited effects on halting disease progression or correcting the underlying pathophysiological mechanisms. Furthermore, COPD is heterogeneous, encompassing numerous subtypes and phenotypes that reflect distinct pathophysiological mechanisms (Zhu et al., 2020).

As hallmark features of COPD, smoking and airflow limitation are not only major risk factors for disease progression (Kapellos et al., 2023), but also frequently the primary drivers of the aforementioned unmet needs. Current standard treatment strategies, including smoking cessation, bronchodilator therapy, pulmonary rehabilitation and vaccination, improve clinical outcomes (Traboulsi et al., 2020; Matsunaga et al., 2019). However, comorbid conditions such as asthma, bronchiectasis and cardiovascular disease are closely associated with COPD’s clinical heterogeneity and may contribute to suboptimal responses to standard therapies (Chen Q. et al., 2025). Furthermore, additional pathogenic mechanisms and potential therapeutic targets remain to be identified, prompting increasing interest in the role of the gut–lung axis in COPD.

Bronchial obstruction is a key point in COPD treatment, and microorganisms are closely associated with the onset and progression of COPD (Nauck et al., 2024; Zhu et al., 2023). Microorganisms are frequently detectable in the airways of COPD patients, both during stable periods and acute exacerbations (Tiew et al., 2022). Isolation of potential pathogenic microorganisms (PPMs) such as *Haemophilus influenzae*, *Streptococcus pneumoniae*, or *Pseudomonas aeruginosa* from respiratory specimens does not meet the definition of colonization, as these microorganisms are associated with tissue damage and inflammatory responses (Feng et al., 2023). Increasing evidence suggests that modulation of the gut microbiota may represent a potential avenue for improving COPD management through the gut–lung axis, although the underlying mechanisms and clinical applications require further investigation and validation.

Recent studies have increasingly focused on the role of gut microbiota in COPD. Emerging evidence indicates that patients with COPD exhibit gut microbiota dysbiosis, characterized by altered microbial composition and reduced microbial diversity (Li et al., 2026), which has been associated with disease severity and progression. Research suggests that gut microbiota and their metabolites are closely associated with the development and progression of various diseases, and their link to pulmonary disorders is referred to as the gut-lung axis (Zhang et al., 2025; Liu et al., 2024). In COPD, gut microbiota may influence disease progression by altering the integrity of the intestinal barrier, producing pro-inflammatory metabolites and modulating bone marrow haematopoietic and immune functions (Wang et al., 2023). Gut microbiota and their metabolites may contribute to the inflammatory and immune responses in COPD (Song et al., 2023), although the precise mechanisms are not fully understood.

Experimental studies further support gut microbiota as a potential therapeutic target in COPD. Experimental studies using faecal microbiota transplantation (FMT) have demonstrated a causal relationship between gut microbiota and COPD, as transplantation of faecal microbiota from COPD patients accelerated disease progression in mice (Jia et al., 2022). FMT from patients with advanced COPD induced COPD-like inflammatory responses, airway remodelling and lung function deterioration in experimental models (Lim et al., 2023). These findings confirm the role of gut microbiota as a pathogenic factor in COPD. Conversely, FMT from healthy donors alleviated pulmonary inflammation and improved lung function in COPD models (Lim et al., 2023). Taken together, these results suggest that gut microbiota may play a role in COPD development and treatment, providing new insights for clinical management and therapeutic approaches.

This review summarizes current evidence regarding gut microbiota dysbiosis in COPD, the underlying mechanisms of the gut–lung axis and emerging microbiota-targeted therapeutic strategies.

## 2 The impact of COPD on gut microbiota

### 2.1 COPD leads to an imbalance in gut microbiota diversity and structure

The diversity and structural imbalance of the gut microbiota in patients with COPD have been extensively studied, particularly in terms of alpha and beta diversity. Studies indicate that, compared to healthy controls, COPD patients exhibit reduced diversity of the gut microbiota and significant structural differences (Li K. et al., 2025; Kotlyarov, 2023). This decrease in diversity may be associated with acute COPD exacerbations (Ma et al., 2021), suggesting that alterations in gut microbiota structure could be linked to them.

In terms of α-diversity, the species richness and evenness of the gut microbiota were both lower in COPD patients than in the control group. β-Diversity analysis revealed compositional differences between COPD patients and healthy individuals, which may reflect variations in the structure of the gut microbiota at an individual level (Ditz et al., 2020). Additionally, changes in the abundance of *Bacteroides species* and a reduction in *Clostridium perfringens* within the gut microbiota of COPD patients may be associated with the onset and progression of the disease (Lai et al., 2024). This dysbiosis may contribute to COPD severity through systemic inflammatory responses (Luo et al., 2025). These findings indicate that gut microbiota dysbiosis is closely associated with COPD development and progression. Similar alterations have also been reported in other chronic inflammatory diseases, particularly inflammatory bowel disease (IBD). In IBD, reduced microbial diversity, depletion of *Firmicutes*, enrichment of Enterobacteriaceae, impaired epithelial barrier function and increased mucosal permeability contribute to persistent intestinal inflammation (Wang et al., 2021; Covello et al., 2024; Pedroza Matute and Iyavoo, 2023). These findings further support the importance of maintaining microbial homeostasis in chronic inflammatory diseases. Reduced gut microbial diversity and depletion of short-chain fatty acid (SCFA)-producing bacteria are common findings in patients with COPD. However, changes in specific bacterial taxa have been less consistent. For instance, studies have reported different patterns in the abundance of *Bacteroides* and *Prevotella* (Li et al., 2026; Wang et al., 2026; Cheng and Zhang, 2024). These variations are likely related to differences in study design, patient characteristics (e.g., stable COPD versus AECOPD), disease severity, sequencing methodologies (16S rRNA sequencing versus shotgun metagenomic sequencing), and data analysis approaches. Therefore, findings on individual bacterial taxa should be interpreted within the context of each study, and future research using standardized protocols and longitudinal designs is needed to better characterize gut microbiota alterations associated with COPD.

### 2.2 Association between clinical phenotypes and gut microbiota in COPD patients

The clinical phenotypes of COPD patients vary according to the severity of their condition and how they present their symptoms. Studies reveal that the status of the gut microbiota of patients in the stable phase of COPD is closely associated with inflammatory markers and pulmonary function (Chenery et al., 2021). Studies have shown that the gut microbiota of COPD patients correlates with the level of airway inflammation (Halpin, 2022). Furthermore, distinct gut microbiota structures are observed in COPD patients between the acute exacerbation phase and the stable phase (Hu et al., 2023). During acute exacerbations, the relative abundance of Firmicutes and Actinobacteria decreases, while that of Bacteroidetes and Proteobacteria increases (Enaud et al., 2023). These findings suggest that disease exacerbation is accompanied by dynamic alterations in gut microbial composition.

The composition of the gut microbiota of COPD patients differs significantly from that of healthy individuals (Kotlyarov, 2022). Studies indicate that COPD patients exhibit characteristic alterations in gut microbial composition, including decreased levels of *Lactobacillus* and *Bifidobacterium* and increased levels of *Enterococcus* and *Prevotella* (Lu et al., 2024). This dysbiosis is closely associated with impaired lung function in COPD patients (Jia et al., 2022). Specifically, the abundance of *Lactobacillus* and *Bifidobacterium* correlates positively with forced expiratory volume in one second (FEV1), FEV1 as a percentage of predicted value (FEV1%pred), and the FEV1/forced vital capacity (FEV1/FVC) ratio. Conversely, the abundance of *Enterococcus* and *Prevotella* correlates negatively with these pulmonary function metrics (Li et al., 2023; Meng et al., 2024). These findings support an association between gut microbial alterations and impaired pulmonary function within the framework of the gut–lung axis.

Patients with COPD frequently experience multiple comorbidities, including cardiovascular disease, diabetes, anxiety and depression (Aldibbiat and Al-Sharefi, 2020). These conditions worsen the patient’s disease state and negatively impact quality of life and prognosis (Park et al., 2024). Research indicates that gut dysbiosis in COPD patients is closely linked to the onset and progression of these comorbidities (Jundi et al., 2023). For example, elderly COPD patients with a high Charlson Comorbidity Index (CCI) are more likely to have gut dysbiosis, suggesting that multiple coexisting diseases can exacerbate intestinal microbial imbalance (Bogdan et al., 2023). This dysbiosis may contribute to the development of these comorbidities through immune and metabolic dysregulation (Mac Aogáin et al., 2023). The composition of the gut microbiota also varies among COPD patients with different comorbidities. For example, COPD patients with diabetes have significantly lower levels of *Bifidobacteria* and *Lactobacilli* in their intestines, while levels of *Enterococcus* and *Escherichia coli* increase (Dahal et al., 2023). This may be related to metabolic disturbances and altered immune function caused by diabetes. Furthermore, COPD patients with cardiovascular disease exhibit markedly reduced gut microbiota diversity, which is associated with elevated inflammatory factor levels (Yu et al., 2024). These studies suggest that different comorbidities may influence the gut microbiota of COPD patients via distinct mechanisms, thereby further impacting disease progression (Table 1).

**TABLE 1:** Gut microbiota alterations in different clinical states of COPD.

Clinical phenotype	Gut microbiota characteristics	Clinical significance	References
Healthy controls	Higher microbial diversity; balanced microbial composition	References microbiota profile	Yang et al. (2026)
Stable COPD	Reduced α-diversity; decreased Lactobacillus and Bifidobacterium; increased Enterococcus and Prevotella	Associated with impaired pulmonary function and chronic systemic inflammation	Jia et al. (2022); Lu et al. (2024)
Acute exacerbation COPD (AECOPD)	Reduced Firmicutes and Actinobacteria; increased Bacteroidetes and Proteobacteria	Associated with enhanced inflammatory responses and disease exacerbation	Enaud et al. (2023)
COPD with diabetes	Reduced Bifidobacterium and Lactobacillus; increased Enterococcus and Escherichia coli	May contribute to metabolic dysregulation and immune imbalance	Dahal et al. (2023)
COPD with cardiovascular disease	Reduced microbial diversity	Associated with elevated systemic inflammatory markers	Yu et al. (2024)

Abbreviations: COPD, chronic obstructive pulmonary disease; AECOPD, acute exacerbation of chronic obstructive pulmonary disease.

### 2.3 COPD drives changes in the gut microbiota

COPD patients frequently exhibit systemic hypoxia and oxidative stress, with markedly elevated levels of oxidative stress markers—such as hydrogen peroxide and eight-isoprostane—in exhaled breath condensate, sputum, and systemic circulation (Wang et al., 2020). This oxidative stress affects not only the lungs, but also multiple organs throughout the body via the circulatory system (Xiang and Luo, 2024), including the gut. Systemic hypoxia, a key pathological feature of COPD, significantly affects the composition and function of the gut microbiota (Shayista et al., 2025). Experimental studies suggest that hypoxic conditions can damage intestinal epithelial cells and increase intestinal permeability (Zhong et al., 2023), thereby disrupting the balance of the gut microbiota. Furthermore, hypoxia may exacerbate dysbiosis by impairing intestinal immune function. Oxidative stress is prevalent in COPD patients, affecting pulmonary inflammation and the gut via the circulatory system (Fekete et al., 2023; Chen Z. et al., 2022). Research indicates that oxidative stress may further disrupt gut microbial homeostasis (Bajinka et al., 2020). Collectively, these changes may further disrupt gut microbial homeostasis.

**FIGURE 1:**
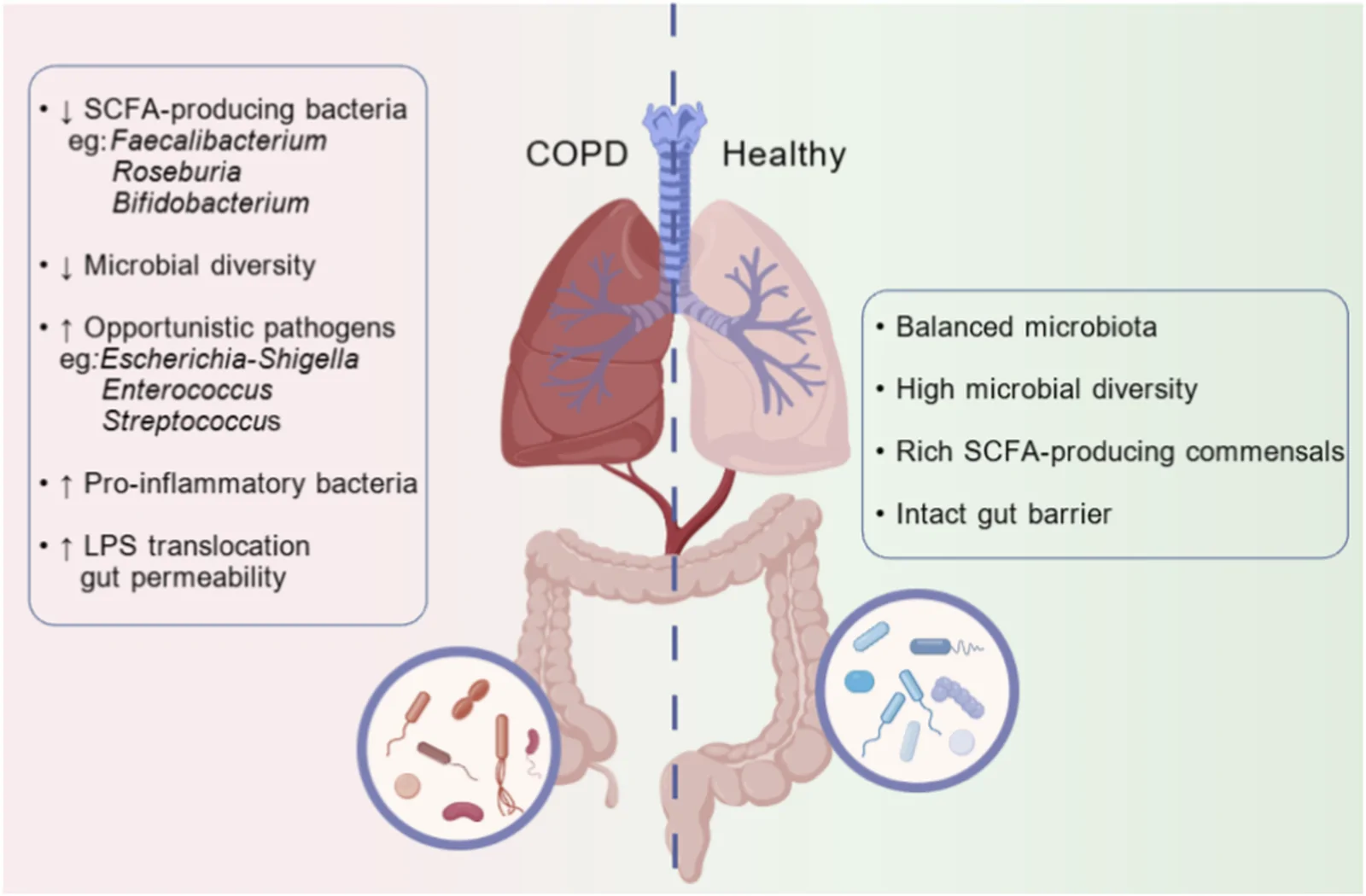
Differences in gut microbiota and gut-lung axis between COPD patients and healthy individuals.

COPD is associated with characteristic alterations in gut microbial composition, accompanied by an increased abundance of opportunistic pathogens and pro-inflammatory bacteria. Compared with healthy individuals, these microbial alterations reflect a disrupted gut microbial ecosystem (Figure 1). Moreover, COPD patients often require multiple medications, including bronchodilators, corticosteroids, and antibiotics (Li L. et al., 2025). While these drugs positively control pulmonary inflammation and improve respiratory function, they may also negatively impact the gut microbiota. For instance, long-term antibiotic use can lead to dysbiosis, increasing the proportion of harmful bacteria and reducing beneficial ones (Wu et al., 2025). Furthermore, glucocorticoid use may alter gut microbiota composition, affecting its diversity and function (Kang et al., 2024). Long-term medication use may influence gut microbial composition through alterations in intestinal homeostasis, immune regulation and microbial metabolism, thereby further aggravating gut microbiota dysbiosis in COPD patients.

In COPD patients, systemic hypoxia, oxidative stress and drug interventions act together to impact the composition and function of the gut microbiota. The gut-lung axis theory offers a useful framework through which to understand this complex relationship, revealing the bidirectional communication mechanisms between the gut and the lungs. Collectively, multiple COPD-associated factors trigger gut dysbiosis, demonstrating an intimate link between COPD and gut microbial ecosystem disruption. Therefore, future research should investigate the interactive mechanisms of systemic hypoxia, oxidative stress and drug interventions. With the regulation of gut microbiota at its core, novel intervention strategies should be developed to improve outcomes for COPD patients, offering new therapeutic targets and approaches for clinical treatment.

The left panel shows the characteristics of the gut in COPD patients. These include a reduction in the number of bacteria that produce SCFAs, such as *Faecalibacterium*, *Roseburia* and *Bifidobacterium*. There is also a decrease in overall microbial diversity, an increase in opportunistic pathogens such as *Escherichia–Shigella*, *Enterococcus* and *Streptococcus*, elevated pro-inflammatory microbiota and impaired intestinal barrier function. This facilitates the translocation of lipopolysaccharide (LPS) and promotes systemic inflammation. In contrast, the right panel depicts a balanced gut microbiome in healthy individuals, characterised by high diversity, an abundance of SCFA-producing bacteria, and an intact intestinal barrier. The illustration highlights the potential role of the gut-lung axis in COPD pathogenesis.

## 3 The impact of gut microbiota on COPD

### 3.1 Immune modulation

The gut microbiota exerts protective immunomodulatory effects on patients with COPD through multiple mechanisms. For example, SCFAs, which are produced by the gut microbiota, exhibit significant anti-inflammatory properties (Chen Y. et al., 2022). These SCFAs can interact with fatty acid receptors (FFARs) on immune cells (Akhtar et al., 2022), thereby suppressing the expression of pro-inflammatory factors such as IL-6, IL-1β and TNF-α. SCFAs may also exert anti-inflammatory effects by inhibiting histone deacetylase complexes and suppressing the nuclear factor kappa B (NF-κB) signalling pathway (van Olst et al., 2021). For instance, a high abundance of *Firmicutes* and *Actinobacteria* in COPD patients is significantly correlated with elevated blood inflammatory markers (e.g., IL-6, IL-8 and TNF-α), whereas a higher abundance of *Bacteroidetes* is associated with improved lung function and lower blood eosinophil counts (Wang et al., 2025). These findings further support the immunomodulatory role of SCFAs in COPD (Li N. et al., 2021). Furthermore, the gut microbiota maintains immune homeostasis by regulating the differentiation and function of immune cells (Song et al., 2020). For instance, certain gut microbiota can modulate the function of regulatory T cells (Tregs), thereby regulating inflammatory responses (Wang et al., 2019). These Tregs are thought to migrate to the lungs, maintaining immune balance and lowering the risk of inflammatory respiratory diseases.

However, gut dysbiosis may also exacerbate inflammatory responses in COPD patients via multiple mechanisms. Imbalanced microbiota can disrupt intestinal barrier function, increasing intestinal permeability and facilitating the translocation of microbial products such as LPS into the circulation, thereby promoting immune activation (Sun et al., 2021; Zhu and Fu, 2024). This systemic inflammation can further intensify chronic inflammation in COPD. Furthermore, dysbiosis modulates immune responses by influencing T-cell differentiation and cytokine production, thereby exacerbating pulmonary inflammation and impairing lung function in COPD patients (Gao et al., 2020). Gut dysbiosis may reduce the anti-inflammatory effects of microbial metabolites, including SCFAs, thereby promoting pulmonary inflammation. Collectively, these findings suggest that gut dysbiosis not only weakens anti-inflammatory signalling but may also amplify pulmonary inflammation through coordinated alterations in microbial metabolites, intestinal barrier integrity and immune regulation.

### 3.2 Regulatory role of gut microbiota metabolites

Gut microbiota-derived metabolites are increasingly recognised as important mediators of COPD pathogenesis. These metabolites including SCFAs, trimethylamine N-oxide (TMAO), tryptophan metabolites and secondary bile acids (SBAs), influence COPD progression by regulating host immune responses and metabolic homeostasis (Okumura and Takeda, 2024; Zhang C. et al., 2021).

SCFAs, primarily acetate, propionate and butyrate, are key fermentation products of dietary fibre by the gut microbiota (Then et al., 2020). These metabolites play a crucial role in regulating intestinal barrier function and immune responses. SCFAs have been shown to exert anti-inflammatory effects by binding to G protein-coupled receptors (GPRs), such as GPR41, GPR43 and GPR109A. This inhibits histone deacetylase (HDAC) activity (Cao et al., 2024). For example, *in vitro* studies have shown that butyrate and propionate can reduce the production of neutrophils stimulated by LPS while decreasing the generation of pro-inflammatory cytokines (e.g., TNF-α and IL-1β) and nitric oxide (NO) (Khudhair et al., 2020). Reduced SCFA availability has been associated with increased disease severity (Li N. et al., 2021), although the immunomodulatory effects of SCFAs may differ according to disease stage, metabolite concentration and host physiological conditions. This may partly explain the inconsistent associations between SCFAs and COPD reported across clinical studies. Differences in dietary intake, patient characteristics, and analytical methods for SCFA quantification are all likely to contribute to the variability in reported findings.

TMAO is a small molecule produced by the liver following the metabolism of choline and L-carnitine by gut microbiota (Nishimoto et al., 2022). TMAO is closely associated with the development of various cardiovascular diseases (CVD) and is also considered a potential risk factor for COPD (Li H. et al., 2022, Ghosn et al., 2023). TMAO may contribute to COPD progression by promoting inflammatory signalling pathways and inflammatory responses (Huang et al., 2021). However, circulating TMAO levels are also influenced by dietary patterns, gut microbial composition and host metabolism, suggesting that elevated TMAO may not exclusively reflect COPD-related pathological changes. This complexity should be considered when interpreting the potential role of TMAO as a biomarker or therapeutic target in COPD. Nevertheless, studies indicate that probiotic supplementation, such as *Lactobacillus acidophilus* (*LGG*), can modulate the gut microbiota and reduce TMAO levels (Gu et al., 2022; Hijová, 2023), thereby mitigating inflammatory responses.

Indole compounds (e.g., indole-3-acetic acid and indole-3-carboxaldehyde), which are produced by the gut microbiota through tryptophan metabolism, play a crucial role in regulating immune responses and intestinal barrier function (Gao H. et al., 2025). These metabolites may alleviate inflammation by binding to the aryl hydrocarbon receptor (AhR) and promoting the secretion of anti-inflammatory cytokines while suppressing pro-inflammatory factors (Poona et al., 2022; Xie et al., 2024). Additionally, indoles indirectly influence metabolic status in COPD patients by enhancing glucagon-like peptide-1 (GLP-1) secretion (Mamic et al., 2021), thereby improving insulin resistance and glucose metabolism. SBAs, such as deoxycholic acid (DCA) and lithocholic acid (LCA), are products of primary bile acid metabolism by gut microbiota (Sanyal et al., 2021; Herlo et al., 2024). These metabolites play a crucial role in regulating lipid metabolism and intestinal barrier function (Hu et al., 2020). SBAs have been proposed to influence intestinal barrier function and immune responses by activating the farnesoid X receptor (FXR) and the G protein-coupled bile acid receptor (TGR5) (Xue et al., 2021).

**FIGURE 2:**
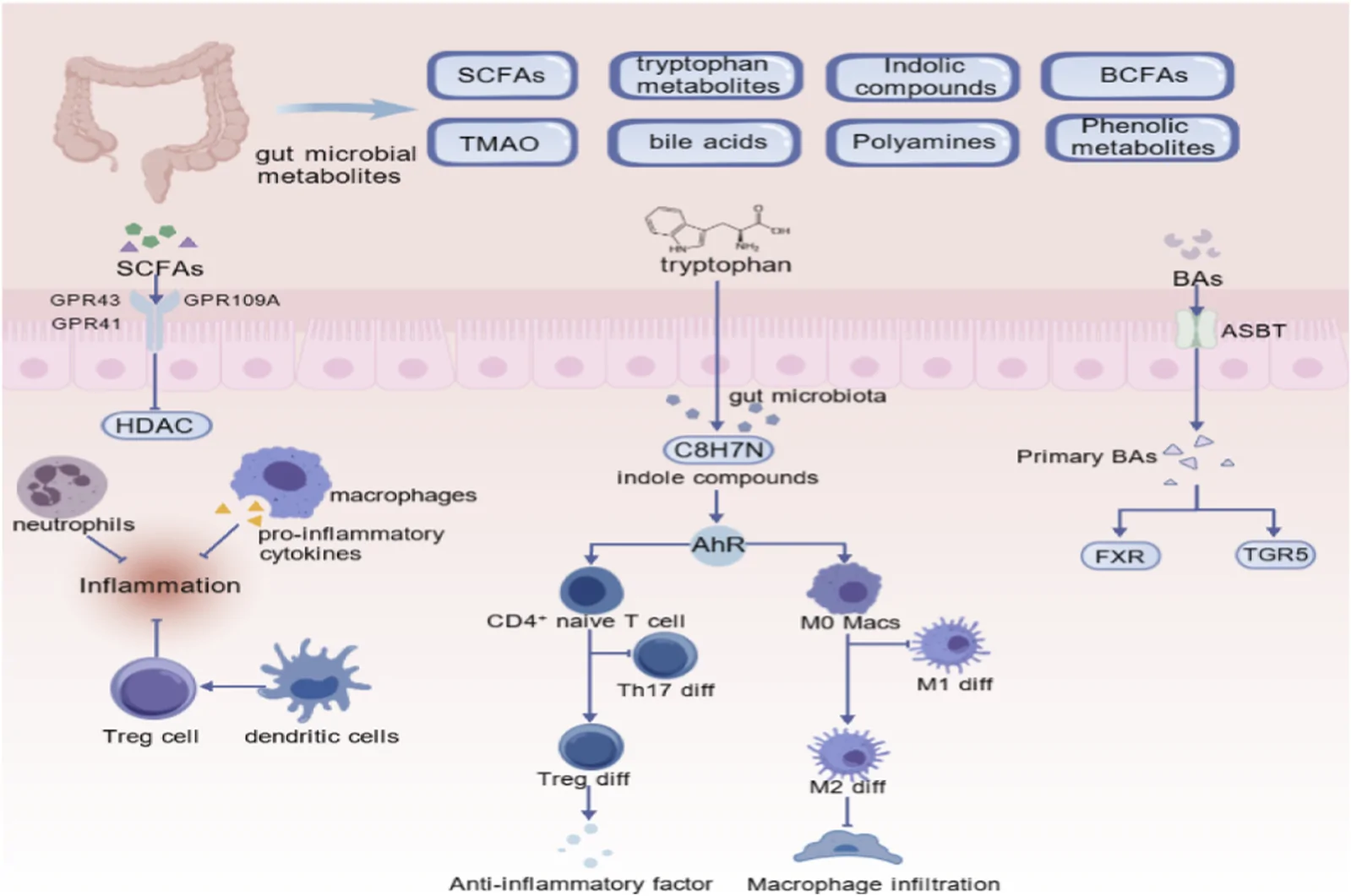
Core mechanisms by which gut microbiota metabolites regulate host immunity and physiological functions.

In patients with COPD, altered levels of SBAs are often closely associated with gut microbiota dysbiosis (Liu T. et al., 2023), and this imbalance may further influence the onset and progression of the disease. Together, the gut microbiota and its metabolites play a key role in regulating the pathophysiology of COPD. Key metabolites, such as SCFAs, TMAO, tryptophan metabolites and SBAs (Li S. et al., 2022; Yang and Wu, 2023), influence disease progression and inflammatory severity by modulating immune responses and metabolic homeostasis (Table 2; Figure 2). Collectively, these metabolites provide important mechanistic links between gut microbiota dysbiosis and COPD progression. Nevertheless, the relative contribution of individual microbial metabolites to COPD progression remains difficult to define because microbial metabolism is influenced by multiple host and environmental factors, and current evidence is derived from studies with heterogeneous designs.

**TABLE 2:** Major gut microbiota-derived metabolites and their potential roles in COPD.

Metabolite	Source	Main targets/pathways	Major biological functions	Potential relevance to COPD	References
SCFAs (acetate, propionate, butyrate)	Produced by gut microbial fermentation of dietary fibre	GPR41, GPR43, GPR109A; HDAC inhibition	Regulate immune responses, maintain intestinal barrier integrity, suppress inflammation	Reduced SCFA availability has been associated with increased disease severity	Li N. et al. (2021); Then et al. (2020); Cao et al. (2024); Khudhair et al. (2020)
TMAO	Produced from microbial metabolism of choline and L-carnitine followed by hepatic oxidation	Inflammatory signalling pathways	Promotes inflammatory responses and metabolic dysregulation	Elevated TMAO levels have been associated with COPD progression	Nishimoto et al. (2022); Huang et al. (2021)
Tryptophan metabolites	Produced by gut microbial tryptophan metabolism	AhR	Regulate immune homeostasis and intestinal barrier function	May alleviate inflammation and contribute to immune regulation	Gao H. et al. (2025); Poona et al. (2022); Xie et al. (2024)
Secondary bile acids (SBAs)	Produced by gut microbial metabolism of primary bile acids	FXR, TGR5	Regulate lipid metabolism, intestinal barrier function and immune responses	May contribute to immune and metabolic homeostasis	Sanyal et al. (2021); Herlo et al. (2024); Hu et al. (2020); Xue et al. (2021)

Abbreviations: SCFAs, short-chain fatty acids; TMAO, trimethylamine N-oxide; HDAC, histone deacetylase; GPR, G protein-coupled receptor; AhR, aryl hydrocarbon receptor; FXR, farnesoid X receptor; TGR5, G protein-coupled bile acid receptor 1; SBAs, secondary bile acids.

The gut microbiota produces a variety of metabolites, such as SCFAs, tryptophan metabolites, indole compounds, branched-chain fatty acids (BCFAs), bile acids, polyamines, phenolic metabolites and TMAO. These metabolites regulate intestinal barrier function and immunity. SCFAs bind to GPR41, GPR43 and GPR109A receptors, inhibit HDAC, modulate inflammatory responses in macrophages and neutrophils, and influence Treg cell differentiation mediated by dendritic cells, thereby exerting anti-inflammatory effects. Tryptophan is metabolised by the gut microbiota into indole compounds which bind to the AhR receptor, thereby regulating the differentiation balance of CD4^+^ naive T cells towards Th17/Treg lineages, as well as the polarisation of M0 macrophages towards M1 (pro-inflammatory)/M2 (anti-inflammatory) states. Primary bile acids, transported by ASBT, participate in the regulatory process via FXR/TGR5 receptors. Together, these pathways demonstrate the critical role of gut microbiota metabolites in maintaining immune homeostasis in the host.

### 3.3 Barrier dysfunction

**FIGURE 3:**
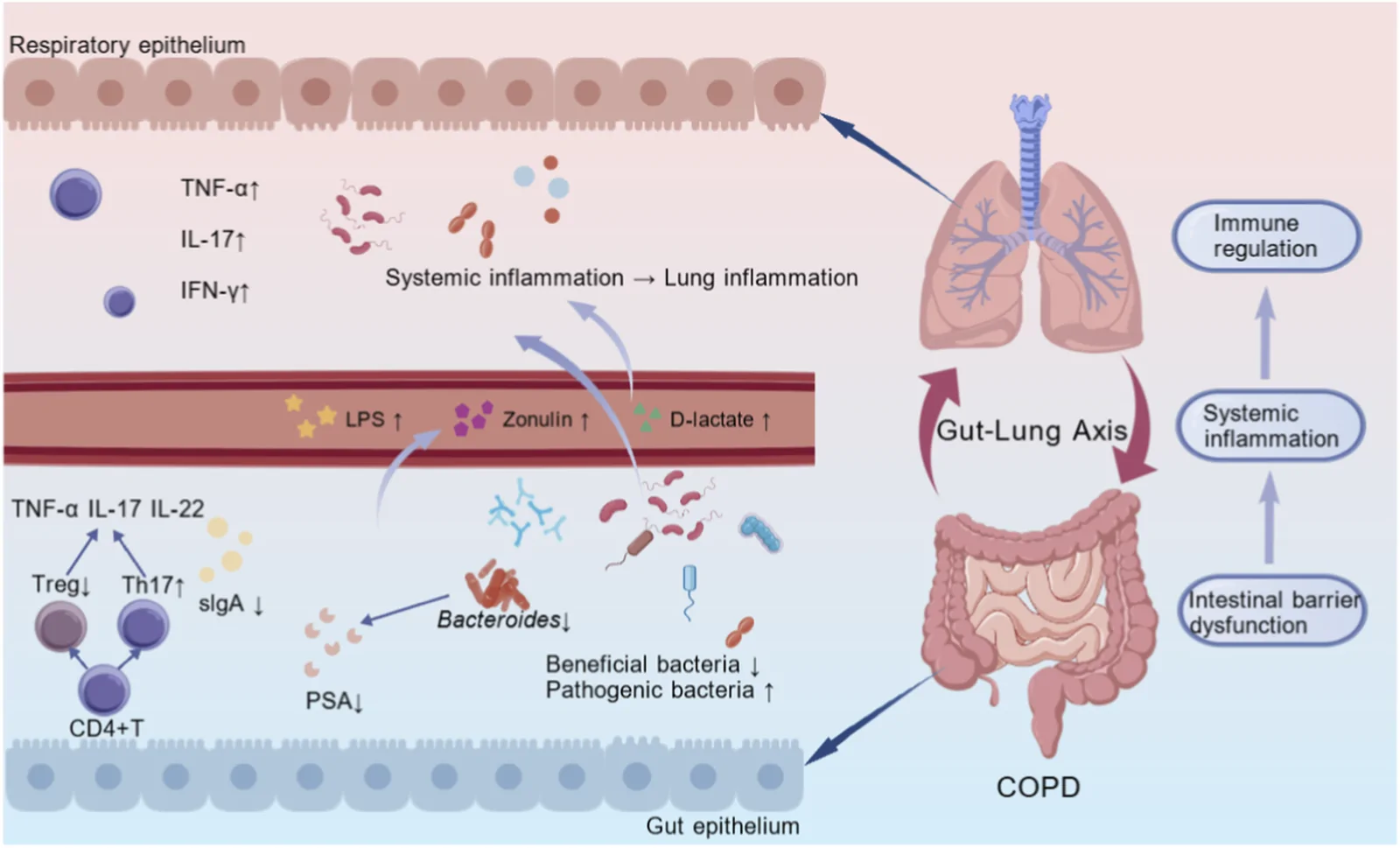
Bacterial translocation and intestinal barrier impairment in chronic obstructive pulmonary disease (COPD).

Patients with COPD often exhibit increased intestinal permeability, which allows gut microbiota and their metabolites to enter the bloodstream and trigger systemic inflammatory responses (Kirschner et al., 2021; Balakin et al., 2025). Gut dysbiosis in COPD is characterised by alterations in microbial composition, which contribute to impaired intestinal barrier integrity (Balderrama-Gómez et al., 2025). This dysbiosis compromises intestinal barrier function and perpetuates and exacerbates pulmonary inflammation by modulating immune responses (Guan et al., 2022). Systemic inflammation arising from intestinal barrier dysfunction affects immune regulation in COPD patients via multiple mechanisms (Figure 3).

A clinical study demonstrated that *Bifidobacterium* therapy significantly improved the composition of the gut microbiota, the function of the gastrointestinal mucosal barrier, and immune function in patients experiencing acute exacerbations of COPD (Song et al., 2024). This suggests that modulating the gut microbiota could effectively improve intestinal barrier function and reduce systemic inflammation in COPD patients. Animal studies indicate that healthy FMT can mitigate emphysema progression by reshaping gut microbial composition and through local and systemic anti-inflammatory effects (Xu et al., 2022; Xiang et al., 2023). These findings further support the role of gut microbiota in maintaining intestinal barrier function. Overall, intestinal barrier dysfunction may represent an important mechanistic link between gut microbiota dysbiosis and COPD progression, as impaired barrier integrity facilitates microbial translocation, systemic inflammation and immune dysregulation.

The dysbiosis observed in COPD patients is characterised by a significant reduction in beneficial bacteria (e.g., *Bacteroides*) and an increase in pathogenic bacteria. This leads to impaired intestinal barrier function. This is accompanied by increased intestinal permeability and elevated zonulin and D-lactate levels, which facilitate the entry of bacterial components and metabolites (e.g., LPS) into the circulation. This triggers systemic inflammation and exacerbates pulmonary inflammation. Alterations in microbial composition also disrupt mucosal immune homeostasis: reduced levels of *Bacteroides* decrease polysaccharide A (PSA) production, thereby affecting the balance of T cells (e.g., Tregs and Th17s). Concurrently, diminished secretory immunoglobulin A (sIgA) levels and reduced antimicrobial peptides weaken local immune defences. Together, these changes promote elevated levels of inflammatory cytokines (e.g., TNF-α, IL-17 and IFN-γ) via the gut-lung axis, thereby exacerbating airway and pulmonary inflammation in COPD patients.

### 3.4 Synergistic effects of environmental and host factors

Environmental and host factors influence not only lung health but also the gut microbiota through the gut-lung axis. Environmental factors such as smoking, diet, ageing, medication and infection interact with host factors including genetic background, immune status and metabolic conditions to shape the gut microbiota of COPD patients (Zhu et al., 2024). Smoking is a primary risk factor for COPD and has been associated with alterations in gut microbial composition, which may contribute to gut dysbiosis and immune dysregulation (Li et al., 2021a; Chen et al., 2023). Gut microbiota also produce various signalling molecules and metabolites (Yuan et al., 2024). Poor dietary habits can also disrupt the balance of the gut microbiome: high-fat, high-sugar Western diets are associated with dysbiosis, whereas fibre-rich diets support microbial equilibrium and health (Chen Z. et al., 2025; Elmhadi et al., 2022). As people age, the diversity of their gut microbes typically declines, which is potentially linked to reduced immune function in the elderly and can further exacerbate COPD pathology (Durack and Lynch, 2019). Meanwhile, the long-term use of antibiotics and glucocorticoids in COPD patients, while effectively controlling pulmonary inflammation, may also disrupt the balance of gut microbiota and induce dysbiosis (Yang-Jensen et al., 2025). Respiratory infections commonly trigger COPD exacerbations (Huang and Shi, 2019). These infections affect not only the lungs but also alter gut microbial homeostasis through gut-lung interactions.

**FIGURE 4:**
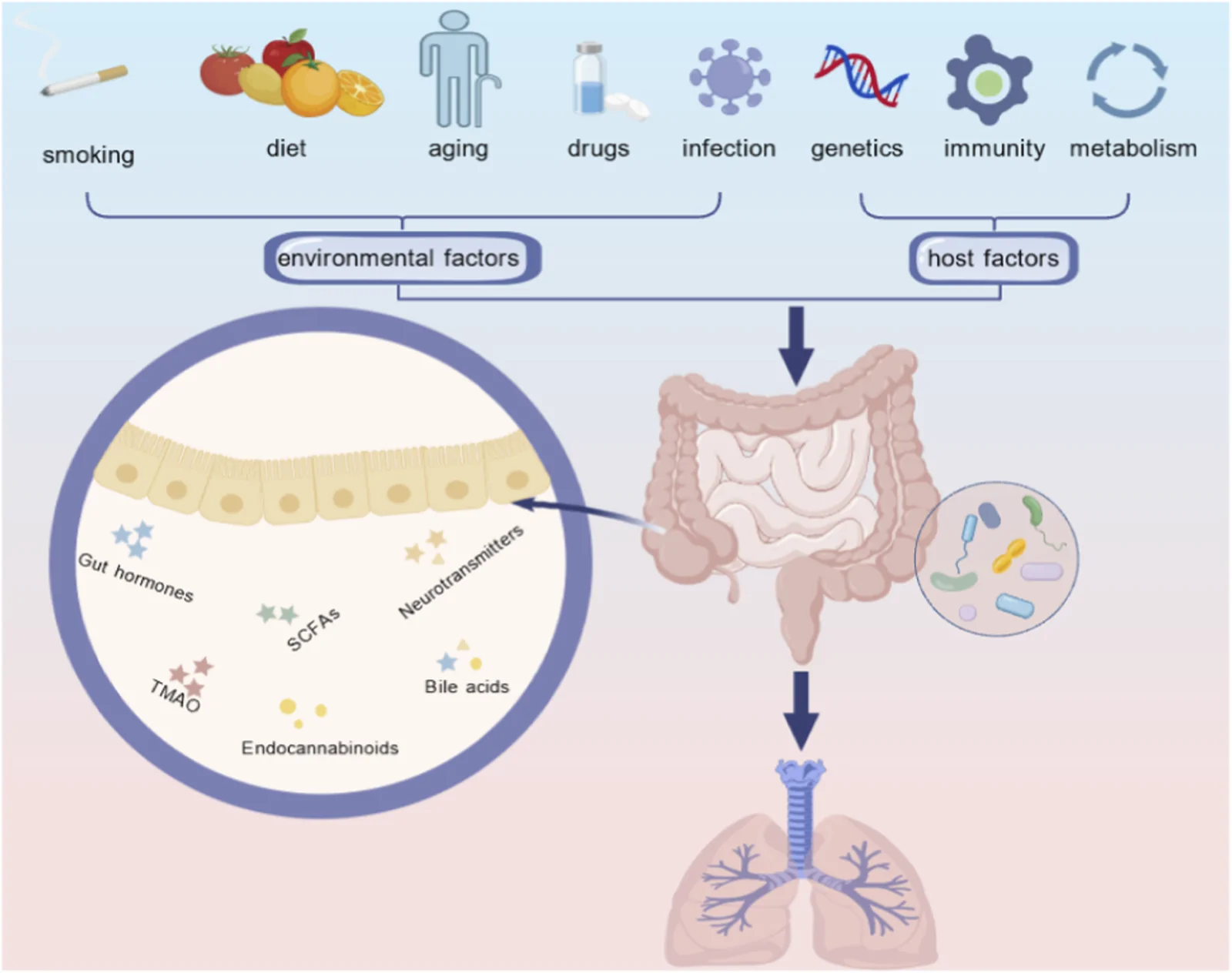
Environmental and host factors jointly shape the gut microbiome and influence lung health via the gut-lung axis.

A person’s genetic background, immune status and metabolic condition can also influence the composition and function of their gut microbiota (Zhang Y. et al., 2021). COPD patients often exhibit immune dysfunction, which may contribute to gut dysbiosis and disease progression (Ye et al., 2023). Furthermore, metabolic disorders such as obesity and diabetes are closely linked to gut microbiota composition (Li et al., 2021b), and may further aggravate COPD pathology by affecting the gut microbiome (Figure 4).

These findings suggest that environmental and host factors do not act independently but may interact through the gut microbiota, collectively influencing immune regulation and inflammatory responses during COPD progression.

The structure and function of the gut microbiota are influenced by environmental factors (such as smoking, diet, ageing, medication use and infection) and host factors (including genetics, immune status and metabolic levels). This microbiota produces a variety of signalling molecules and metabolites, including SCFAs, bile acids, neurotransmitters, enteric hormones, endocannabinoids and TMAO. These substances can influence the physiological and inflammatory states of distant organs, including the lungs, via systemic immune regulatory pathways.

## 4 Gut microbiota-based intervention strategies for COPD

### 4.1 Dietary adjustment

A high-fibre diet is a key strategy for regulating gut microbiota (Bell et al., 2022). Dietary fibre is the main nutrient for intestinal bacteria, which ferment it to produce SCFAs that are active physiologically (Macia et al., 2021; Fan et al., 2021). These metabolites positively affect intestinal function and influence lung health via the bloodstream (Lu et al., 2021). Research indicates a negative correlation between high-fibre diets and COPD incidence, with adequate dietary fibre intake reducing systemic and airway inflammation levels (Brennan et al., 2022; Bailey et al., 2023; Jiang et al., 2023). Furthermore, studies on COPD animal models have demonstrated that high-fibre diets can minimise inflammatory responses and delay the pathological changes associated with emphysema progression (Millares and Monso, 2022).

Although animal studies support the potential benefits of a high-fibre diet in COPD, evidence from randomized controlled clinical trials remains limited.

### 4.2 Probiotics, prebiotics

Probiotics and prebiotics are another effective way of regulating gut microbiota and improving microecological imbalances (Luo et al., 2021). Probiotics are live microorganisms that are beneficial to the host and can directly supplement beneficial bacterial strains, such as *lactobacilli* and *bifidobacteria* (Shen et al., 2024; Gao et al., 2024; Du et al., 2023). Studies suggest that probiotic supplementation can alleviate dysbiosis in COPD patients, lower serum inflammatory marker levels and improve quality of life (Su et al., 2024). Prebiotics, on the other hand, selectively stimulate the growth of beneficial bacteria such as inulin and fructooligosaccharides (Shanmugasundaram Prema et al., 2025). Prebiotic supplementation selectively promotes the growth of beneficial gut microorganisms, thereby supporting microbial homeostasis (Wan et al., 2023).

However, differences in probiotic strains, dosages and treatment protocols, together with the generally small sample sizes of existing studies, make it difficult to compare outcomes across studies and limit the standardization of clinical application.

### 4.3 FMT

Multiple studies using animal models support the potential of FMT in the treatment of COPD. For example, Li *et al.* transplanted faecal microbiota from healthy controls and COPD patients at different stages of the disease into recipient mice. The results showed that the mice that received microbiota from COPD patients exhibited more severe pulmonary inflammation. Following additional smoke exposure, these mice demonstrated impaired lung function, severe emphysematous changes, increased mucus secretion and airway hyperresponsiveness (Li N. et al., 2021). Furthermore, Budden *et al.* utilised a smoke-induced COPD mouse model and found that FMT reduced COPD-associated inflammation and alveolar destruction, improving lung function and alleviating intestinal inflammation and systemic immune dysregulation (Budden et al., 2024). These studies provide a foundation for the application of FMT in COPD treatment.

Although clinical studies of FMT in COPD treatment remain relatively scarce, existing research has yielded positive outcomes. For example, one study found that FMT treatment increased gut microbiota diversity, reduced inflammatory marker levels and improved quality of life in COPD patients (Ye et al., 2025). These preliminary findings suggest that FMT may improve the pathological status and prognosis of COPD patients by reshaping the gut microbiota and mitigating inflammatory responses.

At a mechanistic level, FMT may exert therapeutic effects by restoring gut microbial homeostasis, modulating microbial metabolites and improving intestinal barrier function (Hua et al., 2022; Xie et al., 2025; Tang et al., 2024).

Although FMT has yielded encouraging findings in preclinical and early clinical studies, its clinical application remains limited by the lack of large-scale randomised controlled trials and standardised donor selection and transplantation protocols. Additional studies are required to confirm its long-term efficacy and safety in COPD treatment.

### 4.4 Phage therapy

Bacteriophages are viruses that infect bacteria specifically, and are capable of shaping bacterial communities by inducing lysis of bacterial cells and influencing bacterial phenotypes (Sugai et al., 2023; Lin et al., 2025). Compared to traditional antibiotics, phage therapy has the advantage of being able to target specific bacteria with precision, enabling it to modulate the gut microbiota and eliminate various drug-resistant bacteria (Xu et al., 2023). A significant benefit of this therapy is its specificity towards the targeted bacteria, with minimal interference with other gut microorganisms (Choy and Freedberg, 2020). Phage therapy can be used as an alternative or complementary treatment to antibiotics (Cai et al., 2018).

**FIGURE 5:**
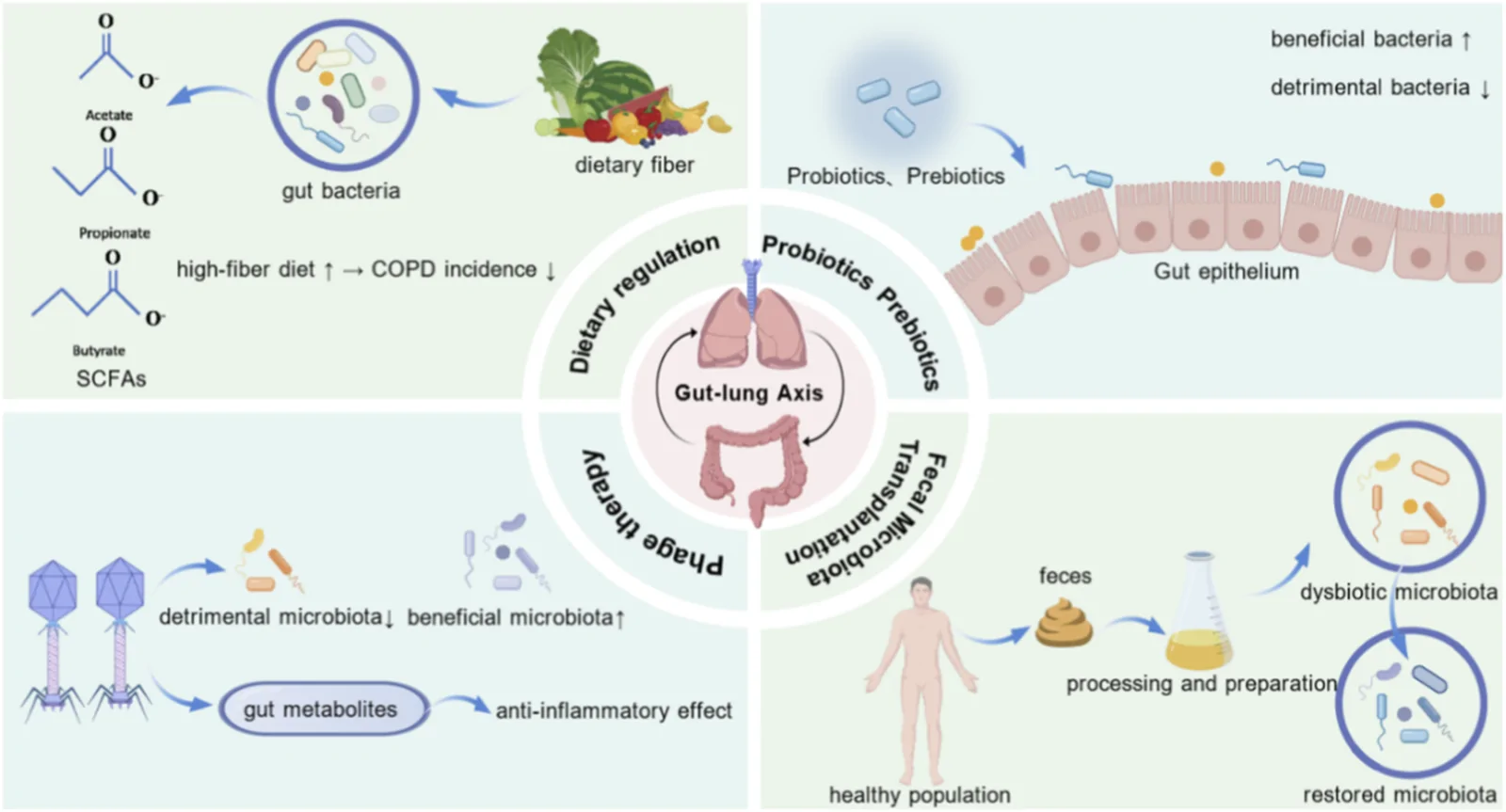
Modulating gut microbiota through multiple intervention strategies to improve COPD.

While research on phage therapy for COPD is limited, existing studies suggest its potential in regulating gut dysbiosis and alleviating inflammation (Bao et al., 2022). For example, studies have found that phage therapy can improve the balance of microorganisms in the gut by reducing the abundance of harmful bacteria while increasing the number of beneficial ones (Febvre et al., 2019). Furthermore, phage therapy may exert anti-inflammatory effects by modulating gut microbial composition and its metabolites, thereby contributing to gut-lung immune homeostasis (Table 3; Figure 5).

**TABLE 3:** Microbiota-targeted COPD interventions: evidence and limitations.

Intervention	Evidence type	Major findings	Main limitations	References
Dietary adjustment (high-fibre diet)	Animal studies	High-fibre diets are associated with a lower risk of COPD and reduced systemic and airway inflammation. Animal studies suggest that dietary fibre supplementation delays emphysema progression	Evidence from randomized controlled trials (RCTs) remains limited	Brennan et al. (2022); Bailey et al. (2023); Jiang et al. (2023); Millares and Monso (2022)
Probiotics and prebiotics	Clinical studies	Probiotic and prebiotic supplementation may improve gut microbiota composition, reduce inflammatory markers and improve quality of life in patients with COPD.	Small sample sizes, heterogeneous probiotic strains, dosages and treatment protocols, and lack of standardized formulations	Luo et al. (2021); Shen et al. (2024); Du et al. (2023); Su et al. (2024)
Faecal microbiota transplantation (FMT)	Animal studies; early clinical studies	FMT reduces pulmonary inflammation and emphysema progression in animal models. Preliminary clinical studies suggest improvements in gut microbiota diversity, inflammatory status and quality of life	Lack of large-scale randomized controlled trials and standardized donor selection and transplantation protocols	Budden et al. (2024); Ye et al. (2025)
Phage therapy	Animal studies; Preclinical studies	Phage therapy may selectively modulate gut microbiota and alleviate inflammation through targeted elimination of pathogenic bacteria	Clinical evidence remains limited, and further studies are required to determine its efficacy, safety and optimal treatment protocols	Xu et al. (2023); Choy and Freedberg (2020); Bao et al. (2022)

Abbreviations: COPD, chronic obstructive pulmonary disease; RCT, randomized controlled trial; FMT, fecal microbiota transplantation.

While phage therapy shows promise in the treatment of COPD, further research is required to evaluate its long-term efficacy and safety. Future research should focus on the following areas: Firstly, more randomised controlled trials should be conducted to validate the efficacy and safety of phage therapy across different stages of COPD progression. Secondly, determining the optimal dosage, administration route and treatment cycle for phage therapy. Third, improving our understanding of its mechanisms of action, particularly its long-term effects on gut microbiota metabolites and immune responses.

The diagram illustrates the pathways through which dietary modification, probiotic/prebiotic supplementation, FMT, and phage therapy exert effects on the gut-lung axis. High-fiber diets, metabolized by gut microbiota to produce short-chain fatty acids (such as acetate, propionate, and butyrate), reduce the risk of developing COPD. Probiotics and prebiotics promote gut homeostasis by enhancing intestinal epithelial barrier function, increasing beneficial bacteria, and reducing harmful bacteria. Fecal microbiota transplantation involves transferring processed functional microbiota from healthy donors into patients’ intestines to restore disrupted gut microbiota and reestablish intestinal microecological balance. Specific phages can be employed to target and eliminate harmful bacteria while enriching beneficial ones in the gut. This process regulates intestinal metabolites and modulates immune status.

## 5 Current challenges and future prospects

### 5.1 Research limitations

Although initial progress has been made in current research on the relationship between gut microbiota and COPD, significant limitations remain. Firstly, many studies have small sample sizes and insufficient statistical power, which can lead to biased results that are difficult to generalise to broader populations. For instance, one study only included 40 COPD patients, which could restrict the generalisability of its findings. Secondly, some studies fail to adequately control for potential confounding factors, such as antibiotic use and glucocorticoid dosage. Such factors are prevalent among COPD patients and can significantly impact the composition and stability of the gut microbiota, thereby hindering accurate assessments of dysbiosis risk. Furthermore, many studies do not distinguish between different COPD phenotypes or disease stages, which can obscure microbial differences across distinct subgroups.

While existing research suggests a strong link between gut dysbiosis and COPD onset and progression, the exact nature of this relationship is unclear. Most current studies remain at the level of correlation analysis and lack experimental validation, which makes it difficult to determine whether gut dysbiosis is a precursor to COPD or a consequence of disease progression. Additionally, the specific mechanisms underlying the gut-lung axis are still in the early stages of research and require further systematic investigation. Secondly, the composition of the gut microbiota is complex and our understanding of the interactions between the gut microbiota and the host is limited. Any localised changes in the gut microbiota may impact host health, but current research methods and technologies are insufficient to fully elucidate these intricate interaction mechanisms.

### 5.2 Frontier directions

Multi-omics technologies integrate data from multiple levels of microbiomics, metabolomics and transcriptomics, providing deeper insights into the complex relationship between the gut microbiota and COPD. For example, metagenomic sequencing revealed significant differences in species composition and abundance between COPD patients and healthy individuals (Bowerman et al., 2020). COPD patients exhibit reduced gut microbial diversity (Gao Y. et al., 2025), with significant enrichment of specific genera, such as Purpureaphilidae and *Veillonella*, and depletion of beneficial bacteria, such as Ruminellaceae and *Bifidobacterium*. These alterations disrupt intestinal microbial balance and may impact lung health via the gut-lung axis.

Metabolomics analysis has also revealed changes in gut metabolites in COPD patients. The study found decreased levels of gut metabolites such as SCFAs (Wallenborn and Vonaesch, 2022), as well as elevated levels of certain metabolites related to inflammation. These metabolic changes may influence the pathological progression of COPD by modulating the host’s immune response (Song et al., 2023; Yang et al., 2022). Transcriptomics research has focused on alterations in host gene expression. The research indicates that the functional activities of gut microbial genes in COPD patients differ significantly from those of healthy controls in terms of biological processes, cellular components and molecular functions (Poto et al., 2022). These altered gene functions may interact with changes in the composition of the gut microbiota to influence the health status of the host.

Advances in AI technology mean that researchers can now use machine learning algorithms to predict the links between characteristics of the gut microbiota and COPD phenotypes. By integrating multi-omics data, AI models can identify the specific microbial features and metabolic biomarkers that are linked to the pathogenesis of COPD. These models enhance our understanding of the complex relationship between gut microbiota and COPD, offering new avenues for personalised treatment. For example, personalised microbial fingerprint profiles generated through machine learning can predict disease progression and treatment response in COPD patients. These predictive models provide clinicians with crucial decision support, helping them to optimise treatment plans and enhance therapeutic outcomes.

Personalised treatment strategies based on gut microbiota, such as probiotics and phage therapy, offer novel approaches to COPD management. Probiotic therapy regulates the balance of gut microbiota by supplementing beneficial strains such as bifidobacteria and lactobacilli (Xu and Wang, 2025; Liu Z. L. et al., 2023; Morniroli et al., 2021; Afrin et al., 2021). Studies indicate that probiotics can improve lung function, reduce inflammatory markers and enhance quality of life in COPD patients (Zhou et al., 2024). Phage therapy restores gut microbiome equilibrium by selectively eliminating harmful bacterial species (Koning et al., 2023; Kazantseva et al., 2023). Compared to antibiotics, phage therapy is more specific and has fewer side effects (Luo et al., 2024). Research suggests that phage therapy has great potential in reshaping the structure of the gut microbiota and alleviating systemic inflammation (Cao et al., 2025).

In summary, multi-omics integration technologies offer new tools for systematically unravelling the intricate mechanisms connecting gut microbiota and COPD. The application of AI technology offers new ways of predicting the links between characteristics of the gut microbiota and COPD phenotypes. The development of personalised probiotic and phage therapies provides new COPD treatment strategies. Further research into the potential applications of these technologies in COPD treatment is needed to provide new scientific evidence and clinical pathways for the precision diagnosis and therapy of COPD.

## 6 Conclusion

Further evidence suggests that the gut microbiota influences the onset and progression of COPD through the gut-lung axis. Patients with COPD often exhibit reduced diversity of gut microbiota, decreased levels of beneficial bacteria and increased levels of pathogenic bacteria, as well as compromised intestinal barrier function. The combined effects of systemic hypoxia, oxidative stress, drug interventions and environmental exposures promote dysbiosis and the translocation of microbial products, thereby exacerbating systemic inflammatory responses. This disrupts pulmonary immune homeostasis and intensifies tissue damage. The gut microbiota and their metabolites, such as SCFAs, TMAO, tryptophan metabolites and SBAs, play a key role in linking gut dysbiosis to immune dysregulation, metabolic disorders and COPD severity.

Despite advances in research, the current understanding remains largely correlational, with significant heterogeneity across studies due to differences in patient populations, disease phenotypes, sequencing methodologies, smoking status, medication exposure and other potential confounding factors. In addition, substantial interindividual variability in gut microbiota composition further complicates the interpretation and comparison of existing findings. Interventions targeting the gut microbiota, including dietary modifications, probiotics, prebiotics, FMT and phage therapy, have demonstrated potential in animal models and early clinical trials, though their efficacy and long-term safety require further validation. In addition, validated biomarkers for identifying patients who are most likely to benefit from microbiota-targeted therapies are still lacking, which further limits their clinical translation. Moreover, most available evidence is derived from cross-sectional studies or animal models, whereas longitudinal and mechanistic human studies remain limited. Future research should integrate multi-omics technologies, advanced computational models, high-quality longitudinal cohort studies and mechanistic human studies to clarify the causal relationship between gut dysbiosis and COPD. A deeper understanding of the gut-lung axis mechanism could lead to more precise intervention strategies based on gut microbiota, providing new theoretical foundations and therapeutic approaches for personalised COPD management and improved prognosis.

## Glossary

COPD: Chronic obstructive pulmonary disease
SCFAs: short-chain fatty acids
TMAO: trimethylamine N-oxide
SBAs: secondary bile acids
FMT: faecal microbiota transplantation
PPMs: potential pathogenic microorganisms
AECOPD: Acute exacerbations of COPD
BALF: bronchoalveolar lavage fluid
IBD: Inflammatory bowel disease
FEV: forced expiratory volume
FVC: forced vital capacity
CCI: Charlson Comorbidity Index
LPS: lipopolysaccharide
FFARs: fatty acid receptors
IL-6: interleukin-6
IL-1β: interleukin-1β
TNF-α: tumor necrosis factor-α
NF-κB: nuclear factor kappa B
Tregs: regulatory T cells
GPRs: G protein-coupled receptors
HDAC: histone deacetylase
NO: nitric oxide
CVD: cardiovascular diseases
LGG: *Lactobacillus* acidophilus
AhR: aryl hydrocarbon receptor
GLP-1: glucagon-like peptide-1
DCA: deoxycholic acid
LCA: lithocholic acid
FXR: farnesoid X receptor
TGR5: G protein-coupled bile acid receptor
BCFAs: branched-chain fatty acids
Th17 diff: Th17 cell differentiation
Treg diff: Treg cell differentiation
Th17: T helper 17 cells
M0 Macs: M0 macrophages
M1 diff: M1 macrophage differentiation
M2 diff: M2 macrophage differentiation
ASBT: apical sodium–dependent bile acid transporter
PSA: polysaccharide A
sIgA: secretory immunoglobulin A
IL-17: interleukin-17
IFN-γ: Interferon-gamma

## References

[B1] Afrin S. Akter S. Begum S. Hossain M. N. (2021). The prospects of Lactobacillus oris as a potential probiotic with cholesterol-reducing property from mother's milk. Front. Nutr. 8, 619506. 10.3389/fnut.2021.619506 33748173 PMC7969506

[B2] Akhtar M. Naqvi S. U. Liu Q. Pan H. Ma Z. Kong N. (2022). Short chain fatty acids (SCFAs) are the potential immunomodulatory metabolites in controlling staphylococcus aureus-mediated mastitis. Nutrients 14 (18), 3687. 10.3390/nu14183687 36145063 PMC9503071

[B3] Aldibbiat A. M. Al-Sharefi A. (2020). Do benefits outweigh risks for corticosteroid therapy in acute exacerbation of chronic obstructive pulmonary disease in people with diabetes mellitus? Int. J. Chron. Obstruct Pulmon Dis. 15, 567–574. 10.2147/COPD.S236305 32214806 PMC7084124

[B4] Bailey M. A. Thompson S. V. Mysonhimer A. R. Bennett J. N. Vanhie J. J. De Lisio M. (2023). Dietary fiber intake and fecal short-chain fatty acid concentrations are associated with lower plasma lipopolysaccharide-binding protein and inflammation. Am. J. Physiol. Gastrointest. Liver Physiol. 324 (5), G369–G377. 10.1152/ajpgi.00176.2021 36791082

[B5] Bajinka O. Tan Y. Abdelhalim K. A. Özdemir G. Qiu X. (2020). Extrinsic factors influencing gut microbes, the immediate consequences and restoring eubiosis. Amb. Express 10 (1), 130. 10.1186/s13568-020-01066-8 32710186 PMC7381537

[B6] Balakin E. Yurku K. Ivanov M. Izotov A. Nakhod V. Pustovoyt V. (2025). Regulation of stress-induced immunosuppression in the context of neuroendocrine, cytokine, and cellular processes. Biol. (Basel). 14 (1), 76. 10.3390/biology14010076 PMC11760489 39857306

[B7] Balderrama-Gómez A. Muñoz-Pérez V. M. Ortiz M. I. Cariño-Cortés R. Castro-Rosas J. Betanzos A. (2025). Using caprylic acid for the prevention and treatment of *Helicobacter pylori* infection and gastric cancer: a review. Metabolites 15 (9), 629. 10.3390/metabo15090629 41003013 PMC12471871

[B8] Bao H. Zhang H. Zhou Y. Zhu S. Pang M. Zhang X. (2022). Dysbiosis and intestinal inflammation caused by Salmonella typhimurium in mice can be alleviated by preadministration of a lytic phage. Microbiol. Res. 260, 127020. 10.1016/j.micres.2022.127020 35462115

[B9] Bell L. Whyte A. Duysburgh C. Marzorati M. Van den Abbeele P. Le Cozannet R. (2022). A randomized, placebo-controlled trial investigating the acute and chronic benefits of American ginseng (cereboost®) on mood and cognition in healthy young adults, including *in vitro* investigation of gut microbiota changes as a possible mechanism of action. Eur. J. Nutr. 61 (1), 413–428. 10.1007/s00394-021-02654-5 34396468 PMC8783888

[B10] Bogdan I. Reddyreddy A. R. Nelluri A. Maganti R. K. Bratosin F. Fericean R. M. (2023). Fungal infections identified with multiplex PCR in severe COVID-19 patients during six pandemic waves. Med. Kaunas. 59 (7), 1253. 10.3390/medicina59071253 PMC10385930 37512065

[B11] Bowerman K. L. Rehman S. F. Vaughan A. Lachner N. Budden K. F. Kim R. Y. (2020). Disease-associated gut microbiome and metabolome changes in patients with chronic obstructive pulmonary disease. Nat. Commun. 11 (1), 5886. 10.1038/s41467-020-19701-0 33208745 PMC7676259

[B12] Brennan M. McDonnell M. J. Harrison M. J. Duignan N. O'Regan A. Murphy D. M. (2022). Antimicrobial therapies for prevention of recurrent acute exacerbations of COPD (AECOPD): beyond the guidelines. Respir. Res. 23 (1), 58. 10.1186/s12931-022-01947-5 35287677 PMC8919139

[B13] Budden K. F. Shukla S. D. Bowerman K. L. Vaughan A. Gellatly S. L. Wood D. L. A. (2024). Faecal microbial transfer and complex carbohydrates mediate protection against COPD. Gut 73 (5), 751–769. 10.1136/gutjnl-2023-330521 38331563

[B14] Cai R. Wu M. Zhang H. Zhang Y. Cheng M. Guo Z. (2018). A smooth-type, phage-resistant *Klebsiella pneumoniae* mutant strain reveals that OmpC is indispensable for infection by phage GH-K3. Appl. Environ. Microbiol. 84 (21), e01585-18. 10.1128/AEM.01585-18 30171001 PMC6193389

[B15] Cao S. Liu M. Han Y. Li S. Zhu X. Li D. (2024). Effects of saponins on lipid metabolism: the gut-liver axis plays a key role. Nutrients 16 (10), 1514. 10.3390/nu16101514 38794751 PMC11124185

[B16] Cao Q. Yang M. Chen M. (2025). Metabolic interactions: how gut microbial metabolites influence colorectal cancer. Front. Microbiol. 16, 1611698. 10.3389/fmicb.2025.1611698 40771692 PMC12325279

[B17] Chen Z. Han S. Zheng P. Zhang J. Zhou S. Jia G. (2022). Landscape of lipidomic metabolites in gut-liver axis of sprague-dawley rats after oral exposure to titanium dioxide nanoparticles. Part Fibre Toxicol. 19 (1), 53. 10.1186/s12989-022-00484-9 35922847 PMC9351087

[B18] Chen Y. Liu Y. Wang Y. Chen X. Wang C. Chen X. (2022). Prevotellaceae produces butyrate to alleviate PD-1/PD-L1 inhibitor-related cardiotoxicity via PPARα-CYP4X1 axis in colonic macrophages. J. Exp. Clin. Cancer Res. 41 (1), 1. 10.1186/s13046-021-02201-4 34980222 PMC8722009

[B19] Chen J. Wang X. Schmalen A. Haines S. Wolff M. Ma H. (2023). Antiviral CD8(+) T-cell immune responses are impaired by cigarette smoke and in COPD. Eur. Respir. J. 62 (2), 2201374. 10.1183/13993003.01374-2022 37385655 PMC10397470

[B20] Chen Q. Wang X. Yao X. Zhang L. Liu X. (2025). COTE and pulmonary comorbidities predict moderate-to-severe acute exacerbation and hospitalization in COPD. Int. J. Chron. Obstruct Pulmon Dis. 20, 1893–1913. 10.2147/COPD.S518218 40524721 PMC12169424

[B21] Chen Z. Jin D. Hu J. Guan D. Bai Q. Gou Y. (2025). Microbiota and gastric cancer: from molecular mechanisms to therapeutic strategies. Front. Cell Infect. Microbiol. 15, 1563061. 10.3389/fcimb.2025.1563061 40529304 PMC12170596

[B22] Chenery A. L. Rosini S. Parkinson J. E. Ajendra J. Herrera J. A. Lawless C. (2021). IL-13 deficiency exacerbates lung damage and impairs epithelial-derived type 2 molecules during nematode infection. Life Sci. Alliance 4 (8), e202001000. 10.26508/lsa.202001000 34127548 PMC8321663

[B23] Cheng Z. X. Zhang J. (2024). Exploring the role of gut-lung interactions in COPD pathogenesis: a comprehensive review on microbiota characteristics and inflammation modulation. Chronic Obstr. Pulm. Dis. 11 (3), 311–325. 10.15326/jcopdf.2023.0442 38563747 PMC11216226

[B24] Choy A. Freedberg D. E. (2020). Impact of microbiome-based interventions on gastrointestinal pathogen colonization in the intensive care unit. Ther. Adv. Gastroenterol. 13, 1756284820939447. 10.1177/1756284820939447 32733601 PMC7370550

[B25] Covello C. Becherucci G. Di Vincenzo F. Del Gaudio A. Pizzoferrato M. Cammarota G. (2024). Parenteral nutrition, inflammatory bowel disease, and gut barrier: an intricate plot. Nutrients 16 (14), 2288. 10.3390/nu16142288 39064731 PMC11279609

[B26] Dahal R. H. Kim S. Kim Y. K. Kim E. S. Kim J. (2023). Insight into gut dysbiosis of patients with inflammatory bowel disease and ischemic colitis. Front. Microbiol. 14, 1174832. 10.3389/fmicb.2023.1174832 37250025 PMC10211348

[B27] Ditz B. Christenson S. Rossen J. Brightling C. Kerstjens H. A. M. van den Berge M. (2020). Sputum microbiome profiling in COPD: beyond singular pathogen detection. Thorax 75 (4), 338–344. 10.1136/thoraxjnl-2019-214168 31996401 PMC7231454

[B28] Du B. Fu Y. Han Y. Sun Q. Xu J. Yang Y. (2023). The lung-gut crosstalk in respiratory and inflammatory bowel disease. Front. Cell Infect. Microbiol. 13, 1218565. 10.3389/fcimb.2023.1218565 37680747 PMC10482113

[B29] Durack J. Lynch S. V. (2019). The gut microbiome: relationships with disease and opportunities for therapy. J. Exp. Med. 216 (1), 20–40. 10.1084/jem.20180448 30322864 PMC6314516

[B30] Elmhadi M. E. Ali D. K. Khogali M. K. Wang H. (2022). Subacute ruminal acidosis in dairy herds: microbiological and nutritional causes, consequences, and prevention strategies. Anim. Nutr. 10, 148–155. 10.1016/j.aninu.2021.12.008 35702144 PMC9168481

[B31] Enaud R. Sioniac P. Imbert S. Janvier P. L. Camino A. Bui H. N. (2023). Lung mycobiota α-Diversity is linked to severity in critically ill patients with acute exacerbation of chronic obstructive pulmonary disease. Microbiol. Spectr. 11 (2), e0506222. 10.1128/spectrum.05062-22 36976010 PMC10100765

[B32] Fan H. Y. Tung Y. T. Yang Y. S. H. Hsu J. B. Lee C. Y. Chang T. H. (2021). Maternal vegetable and fruit consumption during pregnancy and its effects on infant gut microbiome. Nutrients 13 (5), 1559. 10.3390/nu13051559 34063157 PMC8148194

[B33] Febvre H. P. Rao S. Gindin M. Goodwin N. D. M. Finer E. Vivanco J. S. (2019). PHAGE study: effects of supplemental bacteriophage intake on inflammation and gut microbiota in healthy adults. Nutrients 11 (3), 666. 10.3390/nu11030666 30897686 PMC6471193

[B34] Fekete M. Csípő T. Fazekas-Pongor V. Fehér Á. Szarvas Z. Kaposvári C. (2023). The effectiveness of supplementation with key vitamins, minerals, antioxidants and specific nutritional supplements in COPD-A review. Nutrients 15 (12), 2741. 10.3390/nu15122741 37375645 PMC10300814

[B35] Feng S. Yang Y. Wang F. Shi W. Xu J. Tang G. (2023). Low human beta-defensin-2 levels in the sputum of COPD patients are associated with the risk of exacerbations. BMC Pulm. Med. 23 (1), 106. 10.1186/s12890-023-02364-0 37003996 PMC10064533

[B36] Gao S. Chen J. Xie J. Wang J. (2020). The effects of BAFF on T lymphocytes in chronic obstructive pulmonary disease. Respir. Res. 21 (1), 66. 10.1186/s12931-020-01333-z 32160903 PMC7066828

[B37] Gao H. Jiang Y. Zeng G. Huda N. Thoudam T. Yang Z. (2024). Cell-to-cell and organ-to-organ crosstalk in the pathogenesis of alcohol-associated liver disease. eGastroenterology 2 (4), e100104. 10.1136/egastro-2024-100104 39735421 PMC11674000

[B38] Gao H. Sun M. Li A. Gu Q. Kang D. Feng Z. (2025). Microbiota-derived IPA alleviates intestinal mucosal inflammation through upregulating Th1/Th17 cell apoptosis in inflammatory bowel disease. Gut Microbes 17 (1), 2467235. 10.1080/19490976.2025.2467235 39956891 PMC11834480

[B39] Gao Y. Chen L. Zhang J. Wen Z. (2025). CD27 on IgD-CD38-B cells mediates the Coprococcus-COPD link. Int. J. Chron. Obstruct Pulmon Dis. 20, 2173–2182. 10.2147/COPD.S518455 40626314 PMC12232946

[B40] Ghosn B. Onvani S. Ardestani M. E. Feizi A. Azadbakht L. Esmaillzadeh A. (2023). The association between diet quality and chronic obstructive pulmonary disease: a case-control study. BMC Public Health 23 (1), 2071. 10.1186/s12889-023-16586-8 37872531 PMC10591368

[B41] Gu X. Miao Z. Wang Y. Yang Y. Yang T. Xu Y. (2022). New baitouweng decoction combined with fecal microbiota transplantation alleviates DSS-Induced colitis in rats by regulating gut microbiota metabolic homeostasis and the STAT3/NF-κB signaling pathway. BMC Complement. Med. Ther. 22 (1), 307. 10.1186/s12906-022-03766-z 36424592 PMC9686021

[B42] Guan Z. Luo L. Liu S. Guan Z. Zhang Q. Li X. (2022). The role of depletion of gut microbiota in osteoporosis and osteoarthritis: a narrative review. Front. Endocrinol. (Lausanne) 13, 847401. 10.3389/fendo.2022.847401 35418947 PMC8996773

[B43] Halpin D. M. G. (2022). Precision medicine in chronic obstructive pulmonary disease. Chin. Med. J. Engl. 135 (10), 1156–1162. 10.1097/CM9.0000000000002042 35787584 PMC9337263

[B44] Herlo L. F. Salcudean A. Sirli R. Iurciuc S. Herlo A. Nelson-Twakor A. (2024). Gut microbiota signatures in colorectal cancer as a potential diagnostic biomarker in the future: a systematic review. Int. J. Mol. Sci. 25 (14), 7937. 10.3390/ijms25147937 39063179 PMC11276678

[B45] Hijová E. (2023). Benefits of biotics for cardiovascular diseases. Int. J. Mol. Sci. 24 (7), 6292. 10.3390/ijms24076292 37047262 PMC10093891

[B46] Hu H. Lin A. Kong M. Yao X. Yin M. Xia H. (2020). Intestinal microbiome and NAFLD: molecular insights and therapeutic perspectives. J. Gastroenterol. 55 (2), 142–158. 10.1007/s00535-019-01649-8 31845054 PMC6981320

[B47] Hu T. Xu L. Jiang M. Zhang F. Li Q. Li Z. (2023). N6-methyladenosine-methylomic landscape of lung tissues of mice with chronic obstructive pulmonary disease. Front. Immunol. 14, 1137195. 10.3389/fimmu.2023.1137195 37056763 PMC10088907

[B48] Huang Z. Huang Y. Chen J. Tang Z. Chen Y. Liu H. (2022). The role and therapeutic potential of gut microbiome in severe burn. Front. Cell Infect. Microbiol. 12, 974259. 10.3389/fcimb.2022.974259 36467727 PMC9714625

[B49] Huang C. Shi G. (2019). Smoking and microbiome in oral, airway, gut and some systemic diseases. J. Transl. Med. 17 (1), 225. 10.1186/s12967-019-1971-7 31307469 PMC6632217

[B50] Huang R. Yan L. Lei Y. (2021). The gut microbial-derived metabolite trimethylamine N-Oxide and atrial fibrillation: relationships, mechanisms, and therapeutic strategies. Clin. Interv. Aging 16, 1975–1986. 10.2147/CIA.S339590 34876810 PMC8643130

[B51] Jia Y. He T. Wu D. Tong J. Zhu J. Li Z. (2022). The treatment of qibai pingfei capsule on chronic obstructive pulmonary disease May be mediated by Th17/Treg balance and gut-lung axis microbiota. J. Transl. Med. 20 (1), 281. 10.1186/s12967-022-03481-w 35729584 PMC9210581

[B52] Jiang M. Li Z. Zhang F. Li Z. Xu D. Jing J. (2023). Butyrate inhibits iILC2-mediated lung inflammation via lung-gut axis in chronic obstructive pulmonary disease (COPD). BMC Pulm. Med. 23 (1), 163. 10.1186/s12890-023-02438-z 37173731 PMC10182695

[B53] Jundi B. Ahmed H. Reece J. Geraghty P. (2023). The relationship of cholesterol responses to mitochondrial dysfunction and lung inflammation in chronic obstructive pulmonary disease. Med. Kaunas. 59 (2), 253. 10.3390/medicina59020253 PMC9958740 36837454

[B54] Kang J. W. Khatib L. A. Heston M. B. Dilmore A. H. Labus J. S. Deming Y. (2024). Gut microbiome compositional and functional features associate with alzheimer's disease pathology. medRxiv. 10.1002/alz.70417 PMC12221809 40604345

[B55] Kapellos T. S. Baßler K. Fujii W. Nalkurthi C. Schaar A. C. Bonaguro L. (2023). Systemic alterations in neutrophils and their precursors in early-stage chronic obstructive pulmonary disease. Cell Rep. 42 (6), 112525. 10.1016/j.celrep.2023.112525 37243592 PMC10320832

[B56] Kazantseva O. A. Skorynina A. V. Piligrimova E. G. Ryabova N. A. Shadrin A. M. (2023). A genomic analysis of the bacillus bacteriophage kirovirus kirovense Kirov and its ability to preserve milk. Int. J. Mol. Sci. 24 (16), 12584. 10.3390/ijms241612584 37628765 PMC10454425

[B57] Khudhair Z. Alhallaf R. Eichenberger R. M. Whan J. Kupz A. Field M. (2020). Gastrointestinal helminth infection improves insulin sensitivity, decreases systemic inflammation, and alters the composition of gut microbiota in distinct mouse models of type 2 diabetes. Front. Endocrinol. (Lausanne) 11, 606530. 10.3389/fendo.2020.606530 33613446 PMC7892786

[B58] Kirschner S. K. Deutz N. E. P. Jonker R. Olde Damink S. W. M. Harrykissoon R. I. Zachria A. J. (2021). Intestinal function is impaired in patients with chronic obstructive pulmonary disease. Clin. Nutr. 40 (4), 2270–2277. 10.1016/j.clnu.2020.10.010 33873268 PMC8058450

[B59] Koning M. Herrema H. Nieuwdorp M. Meijnikman A. S. (2023). Targeting nonalcoholic fatty liver disease via gut microbiome-centered therapies. Gut Microbes 15 (1), 2226922. 10.1080/19490976.2023.2226922 37610978 PMC10305510

[B60] Kotlyarov S. (2022). Role of short-chain fatty acids produced by gut microbiota in innate lung immunity and pathogenesis of the heterogeneous course of chronic obstructive pulmonary disease. Int. J. Mol. Sci. 23 (9), 4768. 10.3390/ijms23094768 35563159 PMC9099629

[B61] Kotlyarov S. (2023). The role of smoking in the mechanisms of development of chronic obstructive pulmonary disease and atherosclerosis. Int. J. Mol. Sci. 24 (10), 8725. 10.3390/ijms24108725 37240069 PMC10217854

[B62] Lai T. Luo C. Yuan Y. Fang J. Wang Y. Tang X. (2024). Promising intestinal microbiota associated with clinical characteristics of COPD through integrated bioinformatics analysis. Int. J. Chron. Obstruct Pulmon Dis. 19, 873–886. 10.2147/COPD.S436551 38596203 PMC11003469

[B63] Li N. Dai Z. Wang Z. Deng Z. Zhang J. Pu J. (2021). Gut microbiota dysbiosis contributes to the development of chronic obstructive pulmonary disease. Respir. Res. 22 (1), 274. 10.1186/s12931-021-01872-z 34696775 PMC8543848

[B64] Li C. Hou Y. Wang X. Li Y. X. Li F. Zhang C. (2021a). Impact of subthalamic deep brain stimulation on hyposmia in patients with parkinson's disease is influenced by constipation and dysbiosis of microbiota. Front. Neurol. 12, 653833. 10.3389/fneur.2021.653833 33889128 PMC8056012

[B65] Li C. Pi G. Li F. (2021b). The role of intestinal flora in the regulation of bone homeostasis. Front. Cell Infect. Microbiol. 11, 579323. 10.3389/fcimb.2021.579323 33777828 PMC7994858

[B66] Liu C. Li P. Zheng J. Wang Y. Wu W. Liu X. (2022). Role of necroptosis in airflow limitation in chronic obstructive pulmonary disease: focus on small-airway disease and emphysema. Cell Death Discov. 8 (1), 363. 10.1038/s41420-022-01154-7 35973987 PMC9381515

[B67] Li H. Ren M. Li Q. (2022). (1)H NMR-based metabolomics reveals the intrinsic interaction of age, plasma signature metabolites, and nutrient intake in the longevity population in Guangxi, China. Nutrients 14 (12), 2539. 10.3390/nu14122539 35745269 PMC9227029

[B68] Li S. Lin R. Chen J. Hussain R. Zhang S. Su Y. (2022). Integrated gut microbiota and metabolomic analysis reveals immunomodulatory effects of echinacea extract and astragalus polysaccharides. Front. Vet. Sci. 9, 971058. 10.3389/fvets.2022.971058 36118329 PMC9478787

[B69] Li Y. Zou C. Li J. Wang W. Guo Y. Zhao L. (2023). Upper respiratory tract microbiota is associated with small airway function and asthma severity. BMC Microbiol. 23 (1), 13. 10.1186/s12866-023-02757-5 36639753 PMC9837891

[B70] Li K. Liu X. Zhong X. Zeng H. Liu T. Lin B. (2025). Significant associations between high-risk sexual behaviors and enterotypes of gut microbiome in HIV-Negative men who have sex with men. mSphere 10 (7), e0023225. 10.1128/msphere.00232-25 40558044 PMC12306172

[B71] Li L. Liu X. F. Cui Z. F. Zou H. Liu S. L. Wang H. W. (2025). The mechanism of traditional Chinese medicine and natural medicine in treating chronic obstructive pulmonary disease. Int. J. Chron. Obstruct Pulmon Dis. 20, 1249–1266. 10.2147/COPD.S518248 40309575 PMC12042842

[B72] Li J. Zhang H. Zhang P. Hu J. (2026). Potential benefits of gut microbiota modulation in chronic obstructive pulmonary disease. Int. J. Chron. Obstruct Pulmon Dis. 21, 594405. 10.2147/COPD.S594405 42110505 PMC13156967

[B73] Lim E. Y. Song E. J. Shin H. S. (2023). Gut microbiome as a possible cause of occurrence and therapeutic target in chronic obstructive pulmonary disease. J. Microbiol. Biotechnol. 33 (9), 1111–1118. 10.4014/jmb.2301.01033 37164760 PMC10580882

[B74] Lin X. Deng C. Wang L. Shu Y. Li S. Song Y. (2025). Isolation, characterization, and preliminary application of staphylococcal bacteriophages in sichuan paocai fermentation. Microorganisms 13 (6), 1273. 10.3390/microorganisms13061273 40572161 PMC12195502

[B75] Liu T. Guo Y. Liao Y. Liu J. (2023). Mechanism-guided fine-tuned microbiome potentiates anti-tumor immunity in HCC. Front. Immunol. 14, 1333864. 10.3389/fimmu.2023.1333864 38169837 PMC10758498

[B76] Liu Z. L. Chen Y. J. Meng Q. L. Zhang X. Wang X. L. (2023). Progress in the application of Enterococcus faecium in animal husbandry. Front. Cell Infect. Microbiol. 13, 1168189. 10.3389/fcimb.2023.1168189 37600940 PMC10437066

[B77] Liu J. Huang Y. Liu N. Qiu H. Zhang X. Liu X. (2024). The imbalance of pulmonary Th17/Treg cells in BALB/c suckling mice infected with respiratory syncytial virus-mediated intestinal immune damage and gut microbiota changes. Microbiol. Spectr. 12 (6), e0328323. 10.1128/spectrum.03283-23 38727214 PMC11237571

[B78] Lu Y. X. Chang Y. Z. Liang P. Yang C. Q. (2021). Effect of additional Clostridium butyricum on the intestinal flora of chronic hepatitis B patients treated with entecavir. Infect. Dis. Ther. 10 (3), 1519–1530. 10.1007/s40121-021-00463-1 34132991 PMC8322241

[B79] Lu L. Zhu C. Xu J. Hu Y. Dai J. Wang S. (2024). Therapeutic effects of lifei decoction in a murine model of COPD induced by LPS and cigarette smoke. Int. J. Chron. Obstruct Pulmon Dis. 19, 957–967. 10.2147/COPD.S449521 38650680 PMC11034514

[B80] Luo X. Kong Q. Wang Y. Duan X. Wang P. Li C. (2021). Colonization of Clostridium butyricum in rats and its effect on intestinal microbial composition. Microorganisms 9 (8), 1573. 10.3390/microorganisms9081573 34442652 PMC8401576

[B81] Luo X. Liao G. Fu X. Liang H. Niu Y. Lin Q. (2024). A novel and effective therapeutic method for treating Aeromonas schubertii infection in Channa maculata. Anim. (Basel) 14 (6), 957. 10.3390/ani14060957 PMC10967580 38540055

[B82] Luo Z. Liao G. Meng M. Huang X. Liu X. Wen W. (2025). The causal relationship between gut and skin microbiota and chronic obstructive pulmonary disease:a bidirectional two-sample Mendelian randomization analysis. Int. J. Chron. Obstruct Pulmon Dis. 20, 709–722. 10.2147/copd.s494289 40115862 PMC11922780

[B83] Ma N. Qi Y. Liang X. Bai J. Deng J. Li M. (2021). Compare the effect of inhaled corticosteroids and systemic corticosteroids on sputum microbiome of AECOPD. Front. Med. 8, 637246. 10.3389/fmed.2021.637246 PMC7952309 33718410

[B84] Mac Aogáin M. Narayana J. K. Chotirmall S. H. (2023). Reply to Ward et al. Am. J. Respir. Crit. Care Med. 208 (5), 631–632. 10.1164/rccm.202305-0872LE 37348125 PMC10492252

[B85] Macia L. Galy O. Nanan R. K. H. (2021). Editorial: modern lifestyle and health: how changes in the environment impacts immune function and physiology. Front. Immunol. 12, 762166. 10.3389/fimmu.2021.762166 34764963 PMC8576351

[B86] Mamic P. Chaikijurajai T. Tang W. H. W. (2021). Gut microbiome - a potential mediator of pathogenesis in heart failure and its comorbidities: state-Of-The-Art review. J. Mol. Cell Cardiol. 152, 105–117. 10.1016/j.yjmcc.2020.12.001 33307092 PMC7981261

[B87] Matsunaga K. Oishi K. Miravitlles M. Anzueto A. (2019). Time to revise COPD treatment algorithm. Int. J. Chron. Obstruct Pulmon Dis. 14, 2229–2234. 10.2147/COPD.S219051 31631994 PMC6776289

[B88] Meng L. Meng M. Zhang R. Wubulikasimu A. Peng H. Zhang L. (2024). Microbiome-producing SCFAs are associated with preterm birth via trophoblast function modulation. mBio 15 (12), e0270224. 10.1128/mbio.02702-24 39526775 PMC11633107

[B89] Millares L. Monso E. (2022). The microbiome in COPD: emerging potential for microbiome-targeted interventions. Int. J. Chron. Obstruct Pulmon Dis. 17, 1835–1845. 10.2147/COPD.S371958 35983167 PMC9380728

[B90] Morniroli D. Vizzari G. Consales A. Mosca F. Giannì M. L. (2021). Postbiotic supplementation for children and newborn's health. Nutrients 13 (3), 781. 10.3390/nu13030781 33673553 PMC7997220

[B91] Nauck S. Pohl M. Jobst B. J. Melzig C. Meredig H. Weinheimer O. (2024). Phenotyping of COPD with MRI in comparison to same-day CT in a multi-centre trial. Eur. Radiol. 34 (9), 5597–5609. 10.1007/s00330-024-10610-0 38345607 PMC11364611

[B92] Nishimoto Y. Mizuguchi Y. Mori Y. Ito M. Miyazato S. Kishimoto Y. (2022). Resistant maltodextrin intake reduces virulent metabolites in the gut environment: a randomized control study in a Japanese cohort. Front. Microbiol. 13, 644146. 10.3389/fmicb.2022.644146 35602030 PMC9116438

[B93] Okumura R. Takeda K. (2024). The role of the mucosal barrier system in maintaining gut symbiosis to prevent intestinal inflammation. Semin. Immunopathol. 47 (1), 2. 10.1007/s00281-024-01026-5 39589551 PMC11599372

[B94] Park D. Leem J. Lee B. J. Kim K. I. Jung H. J. (2024). Prospective proof-of-concept observational RESEarch about traditional herbal preparation treatment for chronic obstructive pulmonary disease (RESET-COPD-1). Front. Pharmacol. 15, 1437253. 10.3389/fphar.2024.1437253 39391690 PMC11464318

[B95] Pedroza Matute S. Iyavoo S. (2023). Exploring the gut microbiota: lifestyle choices, disease associations, and personal genomics. Front. Nutr. 10, 1225120. 10.3389/fnut.2023.1225120 37867494 PMC10585655

[B96] Poonacha K. N. T. Villa T. G. Notario V. (2022). The interplay among radiation therapy, antibiotics and the microbiota: impact on cancer treatment outcomes. Antibiot. (Basel) 11 (3), 331. 10.3390/antibiotics11030331 PMC8944497 35326794

[B97] Poto R. Loffredo S. Palestra F. Marone G. Patella V. Varricchi G. (2022). Angiogenesis, lymphangiogenesis, and inflammation in chronic obstructive pulmonary disease (COPD): few certainties and many outstanding questions. Cells 11 (10), 1720. 10.3390/cells11101720 35626756 PMC9139415

[B98] Sanyal A. J. Ling L. Beuers U. DePaoli A. M. Lieu H. D. Harrison S. A. (2021). Potent suppression of hydrophobic bile acids by aldafermin, an FGF19 analogue, across metabolic and cholestatic liver diseases. JHEP Rep. 3 (3), 100255. 10.1016/j.jhepr.2021.100255 33898959 PMC8056274

[B99] Shanmugasundaram Prema S. Ganapathy D. Shanmugamprema D. (2025). Prehabilitation strategies: enhancing surgical resilience with a focus on nutritional optimization and multimodal interventions. Adv. Nutr. 16 (4), 100392. 10.1016/j.advnut.2025.100392 39956387 PMC11932842

[B100] Shayista H. Prasad M. N. N. Raj S. N. Prasad A. Lakshmi S. Ranjini H. K. (2025). Complexity of antibiotic resistance and its impact on gut microbiota dynamics. Eng. Microbiol. 5 (1), 100187. 10.1016/j.engmic.2024.100187 40538717 PMC12173824

[B101] Shen X. Xie A. Li Z. Jiang C. Wu J. Li M. (2024). Research progress for probiotics regulating intestinal flora to improve functional dyspepsia: a review. Foods 13 (1), 151. 10.3390/foods13010151 38201179 PMC10778471

[B102] Song X. Liang H. Nan F. Chen W. Li J. He L. (2020). Autoimmune diseases: molecular pathogenesis and therapeutic targets. MedComm 6 (7), e70262. 10.1002/mco2.70262 PMC12171081 40529617

[B103] Song W. Yue Y. Zhang Q. (2023). Imbalance of gut microbiota is involved in the development of chronic obstructive pulmonary disease: a review. Biomed. Pharmacother. 165, 115150. 10.1016/j.biopha.2023.115150 37429232

[B104] Song X. Dou X. Chang J. Zeng X. Xu Q. Xu C. (2024). The role and mechanism of gut-lung axis mediated bidirectional communication in the occurrence and development of chronic obstructive pulmonary disease. Gut Microbes 16 (1), 2414805. 10.1080/19490976.2024.2414805 39446051 PMC11509012

[B105] Su Z. Ma C. Ru X. Zhang S. Wu C. Huang Y. (2024). Effects of probiotic treatment on patients and animals with chronic obstructive pulmonary disease: a systematic review and meta-analysis of randomized control trials. Front. Cell Infect. Microbiol. 14, 1411222. 10.3389/fcimb.2024.1411222 39324058 PMC11422383

[B106] Sugai K. Kawada-Matsuo M. Nguyen-Tra Le M. Sugawara Y. Hisatsune J. Fujiki J. (2023). Isolation of Streptococcus mutans temperate bacteriophage with broad killing activity to S. mutans clinical isolates. iScience 26 (12), 108465. 10.1016/j.isci.2023.108465 38089578 PMC10713843

[B107] Sun Y. Wu D. Zeng W. Chen Y. Guo M. Lu B. (2021). The role of intestinal dysbacteriosis induced arachidonic acid metabolism disorder in inflammaging in atherosclerosis. Front. Cell Infect. Microbiol. 11, 618265. 10.3389/fcimb.2021.618265 33816331 PMC8012722

[B108] Tang B. B. Su C. X. Wen N. Zhang Q. Chen J. H. Liu B. B. (2024). FMT and TCM to treat diarrhoeal irritable bowel syndrome with induced spleen deficiency syndrome-microbiomic and metabolomic insights. BMC Microbiol. 24 (1), 433. 10.1186/s12866-024-03592-y 39455910 PMC11515126

[B109] Then C. K. Paillas S. Wang X. Hampson A. Kiltie A. E. (2020). Association of Bacteroides acidifaciens relative abundance with high-fibre diet-associated radiosensitisation. BMC Biol. 18 (1), 102. 10.1186/s12915-020-00836-x 32811478 PMC7437060

[B110] Tiew P. Y. Thng K. X. Chotirmall S. H. (2022). Clinical aspergillus signatures in COPD and bronchiectasis. J. Fungi 8 (5), 480. 10.3390/jof8050480 PMC9146266 35628736

[B111] Traboulsi H. Cherian M. Abou Rjeili M. Preteroti M. Bourbeau J. Smith B. M. (2020). Inhalation toxicology of vaping products and implications for pulmonary health. Int. J. Mol. Sci. 21 (10), 3495. 10.3390/ijms21103495 32429092 PMC7278963

[B112] van Olst L. Roks S. J. M. Kamermans A. Verhaar B. J. H. van der Geest A. M. Muller M. (2021). Contribution of gut microbiota to immunological changes in alzheimer's disease. Front. Immunol. 12, 683068. 10.3389/fimmu.2021.683068 34135909 PMC8200826

[B113] Wallenborn J. T. Vonaesch P. (2022). Intestinal microbiota research from a global perspective. Gastroenterol. Rep. (Oxf) 10, goac010. 10.1093/gastro/goac010 35419206 PMC8996373

[B114] Wan J. An L. Ren Z. Wang S. Yang H. Ma J. (2023). Effects of galactooligosaccharides on maternal gut microbiota, glucose metabolism, lipid metabolism and inflammation in pregnancy: a randomized controlled pilot study. Front. Endocrinol. (Lausanne) 14, 1034266. 10.3389/fendo.2023.1034266 36777355 PMC9911812

[B115] Wang L. Wang Y. Li H. Feng X. Yuan D. Yang J. (2019). A bidirectional label propagation based computational model for potential microbe-disease association prediction. Front. Microbiol. 10, 684. 10.3389/fmicb.2019.00684 31024481 PMC6465563

[B116] Wang C. Zhou J. Wang J. Li S. Fukunaga A. Yodoi J. (2020). Progress in the mechanism and targeted drug therapy for COPD. Signal Transduct. Target Ther. 5 (1), 248. 10.1038/s41392-020-00345-x 33110061 PMC7588592

[B117] Wang L. Yu M. Yang H. (2021). Recent progress in the diagnosis and precise nanocarrier-mediated therapy of inflammatory bowel disease. J. Inflamm. Res. 14, 1701–1716. 10.2147/JIR.S304101 33953597 PMC8092629

[B118] Wang L. Cai Y. Garssen J. Henricks P. A. J. Folkerts G. Braber S. (2023). The bidirectional gut-lung axis in chronic obstructive pulmonary disease. Am. J. Respir. Crit. Care Med. 207 (9), 1145–1160. 10.1164/rccm.202206-1066tr 36883945 PMC10161745

[B119] Wang Y. Li X. Gao F. (2025). Chronic obstructive pulmonary disease: in-depth analysis of microbiota association and innovative prevention and treatment approaches from the gut-lung axis perspective. Front. Immunol. 16, 1549865. 10.3389/fimmu.2025.1549865 40808958 PMC12343604

[B120] Wang Y. Huang Z. Zheng J. Xu O. Yu H. Ye X. (2026). Profiling of human lung and gut microbiomes in different conditions of chronic obstructive pulmonary disease using ontology-based evidence synthesis and reasoning. Front. Cell Infect. Microbiol. 16, 1771765. 10.3389/fcimb.2026.1771765 42291325 PMC13259719

[B121] Wu J. Liu Y. Liu R. Xiao C. Xuan L. Wu L. (2025). Fishing out AIEC with FimH capturing microgels for inflammatory bowel disease treatment. Nat. Commun. 16 (1), 7924. 10.1038/s41467-025-63276-7 40855058 PMC12378211

[B122] Xu Z. Jiang N. Xiao Y. Yuan K. Wang Z. (2022). The role of gut microbiota in liver regeneration. Front. Immunol. 13, 1003376. 10.3389/fimmu.2022.1003376 36389782 PMC9647006

[B123] Xiang Y. Luo X. (2024). Extrapulmonary comorbidities associated with chronic obstructive pulmonary disease: a review. Int. J. Chron. Obstruct Pulmon Dis. 19, 567–578. 10.2147/COPD.S447739 38476124 PMC10927883

[B124] Xiang W. Xiang H. Wang J. Jiang Y. Pan C. Ji B. (2023). Fecal microbiota transplantation: a novel strategy for treating alzheimer's disease. Front. Microbiol. 14, 1281233. 10.3389/fmicb.2023.1281233 38033557 PMC10687436

[B125] Xie W. Zhong Y. S. Li X. J. Kang Y. K. Peng Q. Y. Ying H. Z. (2024). Postbiotics in colorectal cancer: intervention mechanisms and perspectives. Front. Microbiol. 15, 1360225. 10.3389/fmicb.2024.1360225 38450163 PMC10914944

[B126] Xie H. Zhang H. Zhou L. Chen J. Yao S. He Q. (2025). Fecal microbiota transplantation promotes functional recovery in mice with spinal cord injury by modulating the spinal cord microenvironment. J. Transl. Med. 23 (1), 210. 10.1186/s12967-025-06232-9 39979990 PMC11843963

[B127] Xu Y. Wang M. C. (2025). The intratumoral microbiota in breast cancer: from basic research to clinical translation. Gut Microbes 17 (1), 2560695. 10.1080/19490976.2025.2560695 40948461 PMC12439558

[B128] Xu L. Li Y. He Y. (2023). The variation characteristics of fecal microbiota in remission UC patients with anxiety and depression. Front. Microbiol. 14, 1237256. 10.3389/fmicb.2023.1237256 37744915 PMC10517179

[B129] Xue J. Allaband C. Zhou D. Poulsen O. Martino C. Jiang L. (2021). Influence of intermittent hypoxia/hypercapnia on atherosclerosis, gut microbiome, and metabolome. Front. Physiol. 12, 663950. 10.3389/fphys.2021.663950 33897472 PMC8060652

[B130] Yang Q. Wu Z. (2023). Gut probiotics and health of dogs and cats: benefits, applications, and underlying mechanisms. Microorganisms 11 (10), 2452. 10.3390/microorganisms11102452 37894110 PMC10609632

[B131] Yang H. Qu Y. Gao Y. Sun S. Wu R. Wu J. (2022). Research progress on the correlation between the intestinal microbiota and food allergy. Foods 11 (18), 2913. 10.3390/foods11182913 36141041 PMC9498665

[B132] Yang Y. van Schaik W. McNally A. Zong Z. (2026). Microbiome-mediated colonization resistance to and countermeasures of *Klebsiella pneumoniae* . Nat. Commun. 17 (1), 1761. 10.1038/s41467-026-69690-9 41699406 PMC12913986

[B133] Yang-Jensen S. K. Näegele N. Sonne S. B. Koeninger L. Pineault M. Tremblay F. (2025). Intestinal host-microbe interactions fuel pulmonary inflammation in cigarette smoke exposed mice. Gut Microbes 17 (1), 2519699. 10.1080/19490976.2025.2519699 40581887 PMC12218480

[B134] Ye C. Yuan L. Wu K. Shen B. Zhu C. (2023). Association between systemic immune-inflammation index and chronic obstructive pulmonary disease: a population-based study. BMC Pulm. Med. 23 (1), 295. 10.1186/s12890-023-02583-5 37563621 PMC10416535

[B135] Ye F. Li L. Wang J. Yang H. (2025). Advances in gut-lung axis research: clinical perspectives on pneumonia prevention and treatment. Front. Immunol. 16, 1576141. 10.3389/fimmu.2025.1576141 40330490 PMC12052896

[B136] Yu X. Xiong T. Yu L. Liu G. Yang F. Li X. (2024). Gut microbiome and metabolome profiling in coal workers' pneumoconiosis: potential links to pulmonary function. Microbiol. Spectr. 12 (11), e0004924. 10.1128/spectrum.00049-24 39283109 PMC11537036

[B137] Yuan M. Zhang Z. Liu T. Feng H. Liu Y. Chen K. (2024). The role of nondigestible oligosaccharides in alleviating human chronic diseases by regulating the gut microbiota: a review. Foods 13 (13), 2157. 10.3390/foods13132157 38998662 PMC11241040

[B138] Zhang C. Zhang C. Wang Y. Du M. Zhang G. Lee Y. (2021). Dietary energy level impacts the performance of donkeys by manipulating the gut microbiome and metabolome. Front. Vet. Sci. 8, 694357. 10.3389/fvets.2021.694357 34692802 PMC8531409

[B139] Zhang Y. Chen T. Zhang Y. Hu Q. Wang X. Chang H. (2021). Contribution of trace element exposure to gestational diabetes mellitus through disturbing the gut microbiome. Environ. Int. 153, 106520. 10.1016/j.envint.2021.106520 33774496 PMC8638703

[B140] Zhang Z. J. Gao R. Lu Y. T. Zuo Z. L. Li Y. H. Liu S. (2025). Factors affecting dysbiosis of the gut microbiota in the elderly and the progress of interventions in traditional Chinese and Western medicine. Front. Cell Infect. Microbiol. 15, 1529347. 10.3389/fcimb.2025.1529347 40196043 PMC11973376

[B141] Zhong Y. Wang X. Zhao X. Shen J. Wu X. Gao P. (2023). Multifunctional milk-derived small extracellular vesicles and their biomedical applications. Pharmaceutics 15 (5), 1418. 10.3390/pharmaceutics15051418 37242660 PMC10223436

[B142] Zhou D. He B. Huang Q. Li S. Nan W. Chen Q. (2024). Relationship between dietary live microbe intake and the prevalence of COPD in adults: a cross-sectional study of NHANES 2013-2018. BMC Pulm. Med. 24 (1), 225. 10.1186/s12890-024-03045-2 38724980 PMC11084018

[B143] Zhu Y. Fu Z. (2024). Association of neutrophil-percentage-to-albumin Ratio(NPAR) with depression symptoms in U.S. adults: a NHANES study from 2011 to 2018. BMC Psychiatry 24 (1), 746. 10.1186/s12888-024-06178-0 39468499 PMC11520394

[B144] Zhu T. Li S. Wang J. Liu C. Gao L. Zeng Y. (2020). Induced sputum metabolomic profiles and oxidative stress are associated with chronic obstructive pulmonary disease (COPD) severity: potential use for predictive, preventive, and personalized medicine. Epma J. 11 (4), 645–659. 10.1007/s13167-020-00227-w 33235638 PMC7680486

[B145] Zhu Y. Chang D. (2023). Interactions between the lung microbiome and host immunity in chronic obstructive pulmonary disease. Chronic Dis. Transl. Med. 9 (2), 104–121. 10.1002/cdt3.66 37305112 PMC10249200

[B146] Zhu Q. Qi S. Guo D. Li C. Su M. Wang J. (2024). A survey of fecal virome and bacterial community of the diarrhea-affected cattle in northeast China reveals novel disease-associated ecological risk factors. mSystems 9 (1), e0084223. 10.1128/msystems.00842-23 38108282 PMC10804951

